# A randomized, triple-blinded controlled clinical study with a novel disease-modifying drug combination in equine lameness-associated osteoarthritis

**DOI:** 10.1016/j.ocarto.2023.100381

**Published:** 2023-06-16

**Authors:** E. Skiöldebrand, S. Adepu, C. Lützelschwab, S. Nyström, A. Lindahl, K. Abrahamsson-Aurell, E. Hansson

**Affiliations:** a Department of Biomedical Sciences and Veterinary Public Health, Swedish University of Agricultural Sciences, Uppsala, Sweden; b Department of Clinical Chemistry and Transfusion Medicine, Institute of Biomedicine, Sahlgrenska University Hospital, Gothenburg University, Gothenburg, Sweden; c Hallands Djursjukhus Kungsbacka Hästklinik, Älvsåkers Byväg 20, 434 95 Kungsbacka, Sweden; d Department of Clinical Neuroscience, Institute of Neuroscience and Physiology, The Sahlgrenska Academy, University of Gothenburg, Gothenburg, Sweden

**Keywords:** Osteoarthritis, DMOAD, Companion diagnostics, Biomarkers, Anaesthetic agents, Sildenafil, Glucose

## Abstract

**Objective:**

This study aimed to test a novel treatment combination (TC) (equivalent to sildenafil, mepivacaine, and glucose) with disease-modifying properties compared to Celestone® bifas® (CB) in a randomized triple-blinded phase III clinical study in horses with mild osteoarthritis (OA). Joint biomarkers (reflecting the articular cartilage and subchondral bone remodelling) and clinical lameness were used as readouts to evaluate the treatment efficacy.

**Methods:**

Twenty horses with OA-associated lameness in the carpal joint were included in the study and received either TC (*n =* 10) or CB (*n =* 10) drug intra-articularly-twice in the middle carpal joint with an interval of 2 weeks (visit 1 & 2). Clinical lameness was assessed both objectively (Lameness locator) and subjectively (visually). Synovial fluid and serum were sampled for quantification of the extracellular matrix (ECM) neo-epitope joint biomarkers represented by biglycan (BGN^262^) and cartilage oligomeric matrix protein (COMP^156^). Another two weeks later clinical lameness was recorded, and serum was collected for biomarkers analysis. The overall health status was compared pre and post-intervention by interviewing the trainer.

**Results:**

Post-intervention, SF BGN^262^ levels significantly declined in TC (*P = 0.002*) and COMP^156^ levels significantly increased in CB (*P = 0.002*). The flexion test scores improved in the TC compared to CB (*P =0.033*) and also had an improved trotting gait quality (*P =0.044*). No adverse events were reported.

**Conclusion:**

This is the first clinical study presenting companion diagnostics assisting in identifying OA phenotype and evaluating the efficacy and safety of a novel disease-modifying osteoarthritic drug.

## 1 Introduction

Osteoarthritis (OA) is a whole joint disease with a multifactorial aetiology involving biomechanics, joint overload, low-grade chronic systemic inflammation and immune system activation [1].

The racehorses develop OA spontaneously, therefore can serve as an excellent research animal model and is superior to other induced models [2]. Given their genome similarities with humans, the U.S. Food and Drug Administration (FDA) has approved horses as a translational model to study human OA [3,4].

Currently available pharmacological OA treatments only improve pain or symptoms and are frequently associated with side effects [2]. There are no approved disease-modifying osteoarthritic drugs (DMOADs) on the market yet. A DMOAD typically must show improvement in joint structure, and slow down the progression of cartilage, bone and synovium destruction, with or without an efficacy on joint pain [2,5,6]. Therefore, molecular tools are crucial for diagnosing OA and monitoring intervention efficacy [7].

Both native cartilage oligomeric matrix protein (COMP) and biglycan (BGN) play a structural and functional role in cartilage and bone homeostasis [8,9]. We have previously defined two novel soluble neo-epitopes that reflect ongoing ECM degradation in osteoarthritic articular cartilage and bone. (i) A soluble neo-epitope of biglycan (BGN^262^), elevated in SF from OA horses and positively associated with increased subchondral bone sclerosis (SCBS) [10] (ii) A soluble neo-epitope of cartilage oligomeric matrix protein (COMP^156^), associated with articular cartilage degradation in equine early OA [11,12].

Our proof of concept preclinical research has identified that low concentrations of sildenafil and mepivacaine, when combined with glucose, target basic cellular mechanisms by affecting the inflammatory system [13]. A patented combination of these three substances addressed as treatment combination (TC) in this study exerted an anti-inflammatory effect while restoring Ca^2+^ signalling in gap junction coupled cells [13,14]. We speculate the effect exerted by low concentrations of sildenafil and mepivacaine for OA treatment *in vivo* differs from their normal pharmacological effect.

This study is a randomized, phase III clinical trial, testing TC drug on lame OA horses, with Celeston® bifas® (betamethasone) (CB) as a control substance.

We hypothesised the lameness would improve faster in TC, and the levels of the respective joint biomarkers (BGN^262^ & COMP^156^) in SF and serum should reflect the intervention efficacy. A reduction in SF biomarkers after treatment should indicate a slowdown of the disease progression.

In this study, we have validated BGN^262^ & COMP^156^ for use as companion diagnostics, to identify the OA phenotype and simultaneously monitor the efficacy and safety of a DMOAD in lame horses with mild OA.

## 2 Methods

### 2.1 Study design

The study was performed as a randomized triple-blind (blinded for; the veterinarian assessing the outcome, the horse owners/trainers, researchers and the statistician) phase III trial with a parallel group design and equal allocation ratio, and was approved by the Ethics Committee, Lund, Sweden (D.nr:5.8.18–06590/2020) and the Swedish Medical Product Agency, Uppsala, Sweden (D.nr 5.1-2020-31501). All horse owners/trainers signed a study consent and were allowed to opt-out any time without providing a reason. A computer-generated randomization list was made by the statistician Claudia von Brömssen (SLU, Uppsala, Sweden) using the interactive webpage ‘Randomization table for clinical trials (https://aurora.shinyapps.io/random_gen/)! by A. Baluja.

The CONSORT (Consolidated Standards of Reporting Trials) checklist has been used when reporting the clinical trial 2010 [15].

### 2.2 Sample size

A sample size calculation was performed with a 95% confidence level and 80% power, considering a 20% reduction in COMP^156^ levels in SF after treatment (based on an earlier unpublished pilot study). From there, it was intended to include a total of 10 horses in each study group.

### 2.3 Inclusion and exclusion criteria

Standardbred trotters (STBs) (age: 2–9 years), with lameness originating from the carpal joint, were recruited at Kungsbacka Horse Clinic, Kungsbacka, Sweden. Inclusion criteria were a positive response to diagnostic intra-articular anaesthesia of the affected carpal joint and mild radiographic signs of SCBS (only mild bone sclerosis in sky projection were accepted) with no remodelling of the affected joint. Horses that received intra-articular medications such as corticosteroids or hyaluronic acid within the previous three to six months, were excluded. Bilateral carpal lameness was accepted. The data collected throughout the trial was logged individually for each horse.

### 2.4 Visit 1- clinical examination and treatments

At visit 1, the horses were subjected to a complete lameness examination (by veterinarian 1). The initial lameness was evaluated objectively with Lameness locator® and subjectively following the flexion test by visual grading of lameness (scale 0–5) (suppl.Table 1). Horses fulfilling inclusion criteria were assigned to the respective treatment group according to randomization list by the assisting veterinarian (veterinarian 2).

The products were kept in a locker at the clinic. Horses in TC group received 5 ​ml ​TC, a combination of Carbocain® (mepivacaine hydrochloride, 20 ​mg/ml) solution, (AstraZeneca, Södertälje, Sweden), Revatio® (sildenafil, 0.8 ​mg/ml) solution (Pfizer, Bruxelles, Belgium) and glucose solution (50 ​mg/ml) (Braun Melsungen, Melsungen, Germany) mixed in sterile water (Braun Melsungen, Melsungen, Germany). The formulation concentrations of the individual drug components are patented (Patent application number 185133–3). CB group received 0.5 ​ml of 5.7 ​mg/ml CB (Celestone® bifas® (betametason) solution (Organon, Oss, The Netherlands). Both drugs were injected into the affected upper and middle carpal joint.

Injections were administered the same day horses arrived at the clinic, after confirming eligibility, source of lameness and evaluating the radiographs. Treatments were double-blinded except for the assisting veterinarian who prepared the syringes according to the randomization list and administered the injections. Owners remained blinded during the whole study period and the veterinarians until after the statistical analyses were performed.

### 2.5 Outcomes and follow-up

Lameness evaluation (objectively and subjectively) (see suppl. data material-methods and suppl.Table 1).

### 2.6 Sampling of SF and serum for biomarkers

SF samples were collected from both left and right forelimb carpal joint (middle and upper joint compartments) before administering the local anaesthetic at visits 1 and 2 and serum samples at visits 1, 2 and 3 (sample handling described in suppl. data).

### 2.7 Rehabilitation after visit 1

Post-first treatment, the horses were allowed to rest in a box, thereafter, were allowed 30 ​min hand-walking per day with free access to a small paddock during the first week. During second week, they were allowed 60 ​min hand-walking per day.

### 2.8 Visit 2 after 14 days

After 14 days, the evaluating veterinarian performed a second lameness evaluation on horses with Lameness Locator® and flexion. SF and serum sampling were performed as described earlier. After the second clinical examination, the double-blinded trial was continued by injecting a second intra-articurlar treatment by the attending veterinarian.

### 2.9 Rehabilitation after visit 2

Post-second treatment, the rehabilitation was same as in visit 1 for a week and thereafter the horses were allowed to jogg and trot during the second week.

### 2.10 Visit 3 after 28 days

After 28 days, the veterinarian performed a third lameness evaluation and serum was collected.

### 2.11 Interview at visit 1 and follow-up after 60 days (visit 4)

The trainers (professionals and amateurs) were interviewed with the questionnaire (suppl. data).

### 2.12 Diagnostic accuracy for biomarkers

The STARD (Standards for Reporting of Diagnostic Accuracy Studies) checklist has been used to report the diagnostic part of the clinical trial [16].

Custom-made ELISAs used for BGN^262^ and COMP^156^ neo-epitope quantification were previously developed and validated for serum and SF in horses [[10], [11], [12]] (suppl.data).

BGN^262^ lower and higher detection levels were 1.95 and 2000 ​ng/ml, respectively with intra-assay variation >11%, and inter-assay variation ≥6%. The area under the ROC curve (AUC) was 0.957 (95% confidence interval 0.868 to 1.000; p= 0.0004). COMP^156^ lower and higher detection levels were 0.156 μg/ml and 2000 μg/ml, respectively with intra-assay variation >8%, and inter-assay variation ≥6%. The area under the ROC curve was 0.99 (standard error, 0.0006) (95% confidence interval 0.99–1.00, p<0.0001).

As reference values for a healthy horse, BGN^262^ was set to (mean ​± ​sd) 173±92 ​ng/ml [10] and COMP^156^ to 16.5±5.9 ​μg/ml [11].

### 2.13 Safety data

Full blood profile for drug safety were analysed at visits 1, 2 and 3.

### 2.14 Anamnestic evaluation of clinical side effects

During visits 2 and 3, trainers were asked if the horses had experienced any side effects, such as swelling of the joints or flares following the treatment.

### 2.15 Study outcomes

The study was blinded until the primary and secondary outcomes were analysed. The code was broken after the full statistical analysis was reported.

### 2.16 Primary outcomes

BGN^262^ & COMP^156^ concentration change in the carpal joint (upper or middle joint compartment) with the highest value at visit 1 vs visit 2.

In parallel to this study, our group has validated BGN^262^ as a bone marker [10]. Therefore, the BGN^262^ quantification has been included in primary outcomes through an amendment sent to the Medical product agency, Uppsala, Sweden.

### 2.17 Secondary outcomes

BGN^262^ & COMP^156^ concentration change in the middle carpal joint compartment at visit 1 vs visit 2.

Comparison of the number of non-lame (lameness ​= ​0 after flexion test) horses at both visits 2& 3.

Blood sample analysis reports.

### 2.18 Exploratory results

Lameness Locator (Q score) between visits 1, 2 & 3.

BGN^262^ & COMP^156^ concentration change in the carpal joint (upper or middle joint compartment) with lowest value at visit 1 vs visit 2.

The change in lameness score post flexion test between the visits (1, 2 & 3).

The change in BGN^262^ & COMP^156^ serum concentrations between the visits (1, 2 & 3).

### 2.19 Longterm follow-up results

Interview with the trainers.

### 2.20 Statistical analysis

Assessment of the normal distribution assumption for biomarkers concentrations (BGN^262^ and COMP^156^ in SF) were made for both non-logarithmic and logarithmic values. The normal distribution assumption was evaluated in two ways: 1) analysing the distribution of the error terms for linear mixed models. 2) analysing the distribution for differences in concentration between visits 1 and 2.

Shapiro Wilk's test was made for both cases. In short, the analyses show that data deviate from the normal distribution, which means that a non-parametric method was primarily chosen to perform the analyses on the concentration of the biomarkers in SF.

The statistical methods used to replace linear mixed models were the Wilcoxon signed rank test and the Wilcoxon rank sum test. For these methods, the same results were obtained for logarithmic and non-logarithmic values, as the tests were rank based. Estimates and CI for original (non-logarithmic) values are also presented. BGN^262^ and COMP^156^ values for the individual horses are presented as the mean of the duplicates.

For the follow-up interview questions, Fisher's exact test was used. All results are presented as mean ​± ​SD (demographic data and Q-score), for biomarker data 95% confidence interval [CI]. A significance level of 5% was used. R (version 4.0.0) has been used for all analyses (full report in suppl. data).

### 2.21 Study limitations

At visit 3 the SF was not sampled because most of the horses were sound (due to ethical reasons and risk for intra-articular infection). Therefore, the SF COMP^156^ and BGN^262^ data are not available for this visit.

### 2.22 Data availability

The study data generated and recorded during the trial is not publicly available and resides with the corresponding author (E.S) and can be provided upon request.

## 3 Results

**Fig. 1:**
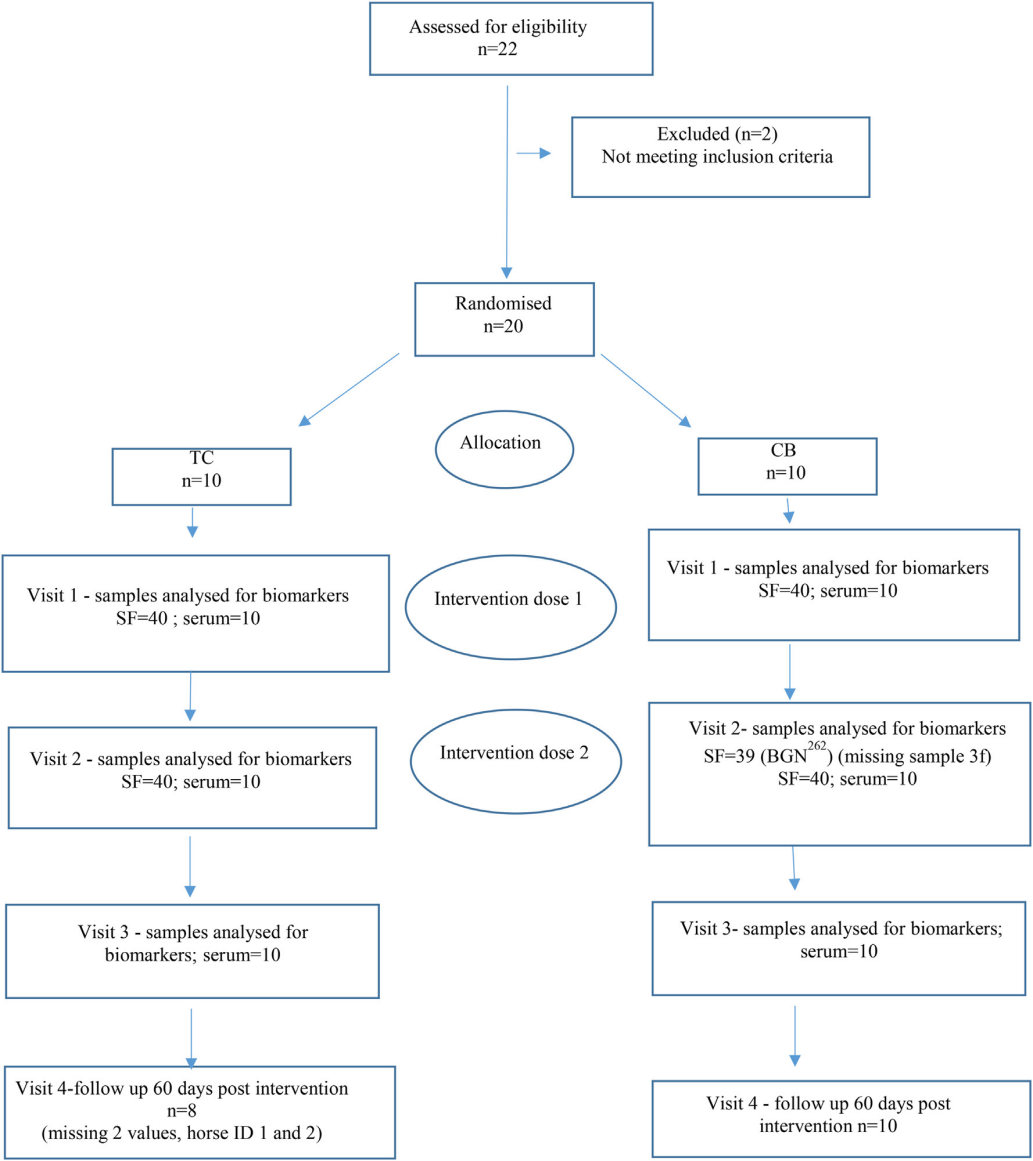
Trial overview. Twenty horses completed the study and were included in the analysis.

Twenty STBs fulfilling the inclusion criteria were included in the study between December 2020 to December 2021 (Fig. 1). For demographic and baseline data see Table 1.

**Table 1:** Demographic data and baseline values (mean±sd) for the horses in TC and CB included in the trial. *TC= treatment combination, CB=Celeston® Bifas®.*

	TC (N=10)	CB (N=10)
Age	3.50 (1.84)	4.50 (2.37)
Sex: Mare, n (%)	2 (20)	1 (10)
Sex: Stallion, n (%)	4 (40)	4 (40)
Sex: Gelding, n (%)	4 (40)	5 (50)
Lameness post-flexion test visit	1.50 (0.41)	1.40 (0.39)
Initial lameness Q-score visit 1	13.61 (5.74)	10.67 (9.27)
Carpal joint treated (right/left)	9/1	3/7
BGN^262−^synovial fluid (ng/ml)	518.8 (133)	465.8 (195)
BGN^262−^serum (ng/ml)	1526 (188)	1477 (260)
COMP^156−^synovial fluid (ug/ml)	26.0 (12.3)	25.8 (13.9)
COMP^156−^serum(ug/ml)	6.7(2.0)	6.5 (2.5)

### 3.1 Clinical lameness

At the inclusion, the baseline lameness score with Lameness Locator® (Q-score) and flexion did not differ between the groups (suppl.Table 6). Lameness post flexion test at visits 1,2 and 3 for the individual horses are also reported (suppl.Table 2).

**Fig. 2:**
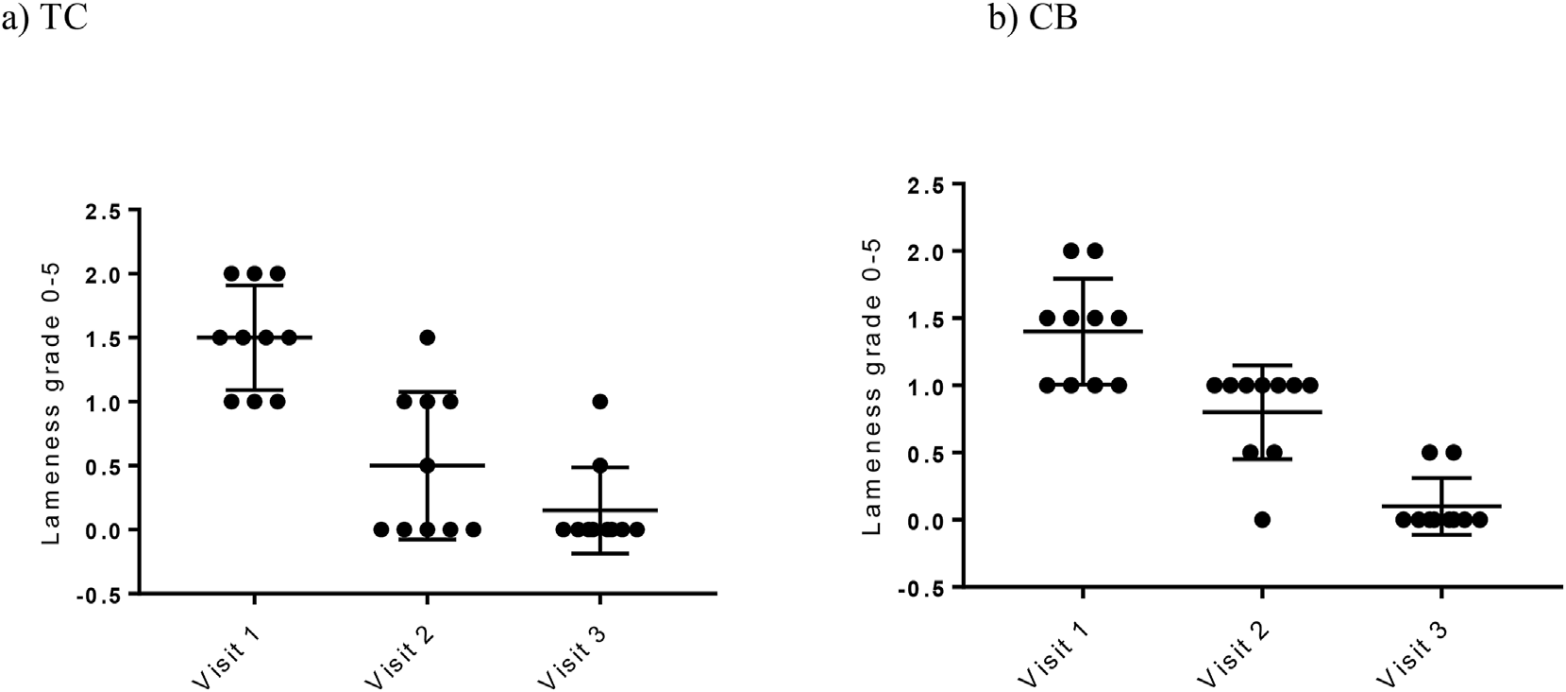
Clinical lameness after flexion test at visits 1, 2 and 3 for horses in a) TC (n=10) and b) CB (n=10). There was a statistically significant decrease in lameness grade at visit 2 for horses in the TC compared to the CB group (p = 0.033). TC= treatment combination, CB= Celeston® Bifas®. a) TC b) CB

TC became free of lameness faster (score 0) from visit 1 to visit 3, compared to CB (*p = 0.033*). In both groups at visit 3, there were two non-responders (Fig. 2).

Biomarkers concentrations of COMP^156^ and BGN.^262^

The SF biomarkers concentrations did not differ between the upper or middle compartment of the carpal joint hence middle carpal joint results were used.

### 3.2 Biomarker levels at inclusion

The SF BGN^262^ levels were above the reference range (≥265 ​ng/ml) for all horses except one (in CB). For COMP^156^, (TC: n=6 and CB: n=5), the concentrations were above the reference value (≥22.4 ​μg/ml).

### 3.3 Biomarker levels used for measuring treatment efficacy and safety

#### 3.3.1 BGN^262^

At visit 1, the SF BGN^262^ levels were 519 ​ng/ml [424–614] in TC and 466 ​ng/ml [327–605] in CB. At visit 2, the concentration declined to 269 ​ng/ml [187–351] in TC and increased to 1545 ​ng/ml [−48- 3138] in CB.

**Fig. 3:**
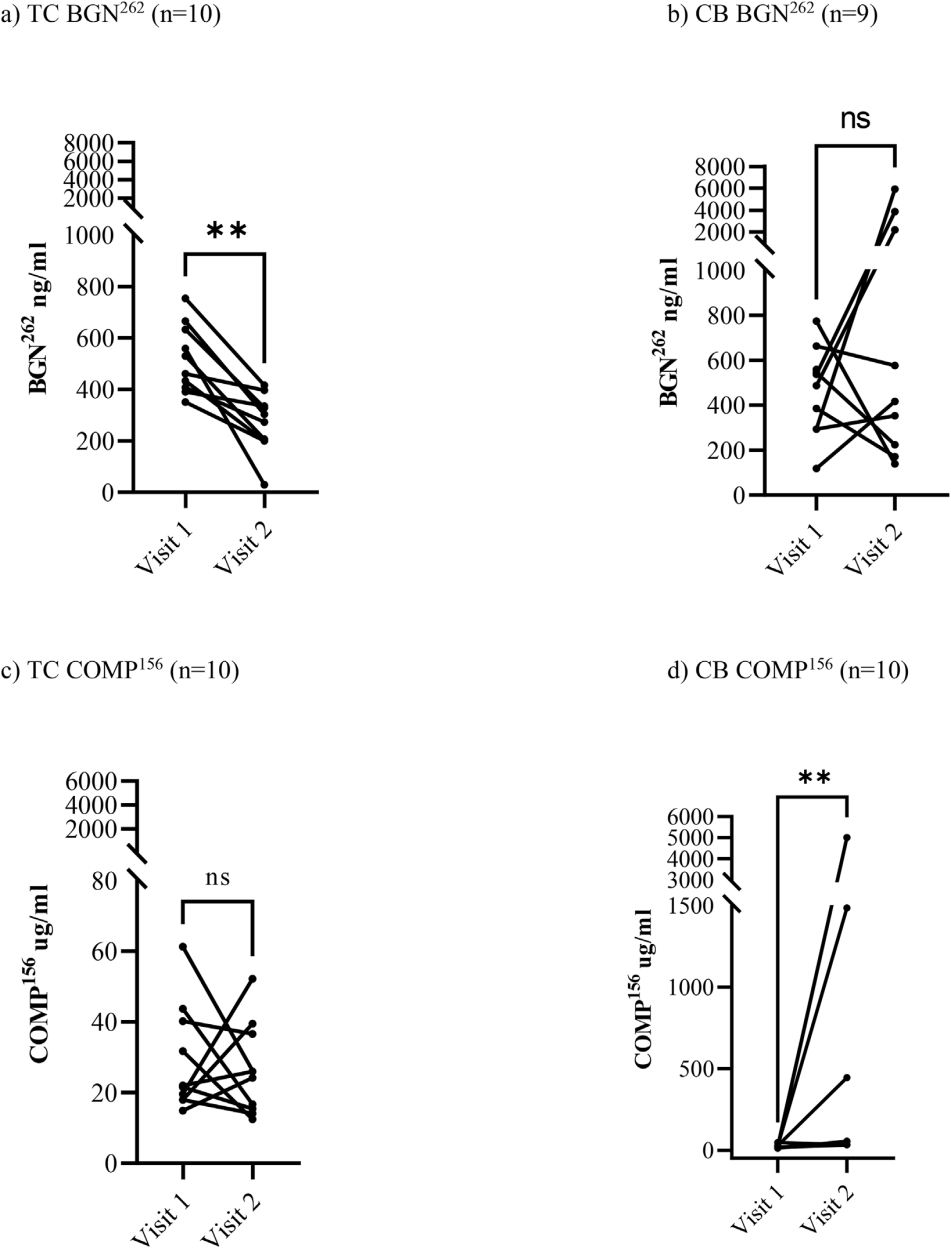
Concentration of BGN^262^ (ng/ml) and COMP^156^ (μg/ml) in synovial fluid (middle carpal joint) at visit 1 and after treatment at visit 2 for horses in TC and CB. There was a reduction in a) BGN^262^ for TC at visit 2 (p= 0.002) unlike in the b) CB group. No reduction in COMP^156^ in c) TC, however an increase in COMP^156^ for d) CB at visit 2 (p= 0.0069). TC= treatment combination, CB= Celeston® Bifas®

At visit 2, TC showed a significant decrease in SF BGN^262^ levels (*p= 0.002*) which was not found in CB. Instead, the latter showed an increase in BGN^262^ concentration, however, not statistically significant (Fig. 3a and b) (Table 2).

**Table 2:** Concentration of BGN^262^ (ng/ml) and COMP^156^ (μg/ml) in SF from middle carpal joint at visit 1 and 2 for TC and CB group. As a reference, the value for healthy horses BGN^262^ ​≤ ​265 ​ng/ml (173±92) [10] and for COMP^156^ ​≤ ​22.4 ​μg/ml (16.5±5.9)^11^. TC= treatment combination, CB=Celeston® Bifas®.

Horse IDs TC	BGN^262^ visit 1	BGN^262^ visit 2	COMP^156^ visit 1	COMP^156^ visit 2	Horse IDs CB	BGN^262^ visit 1	BGN^262^ visit 2	COMP^156^ visit 1	COMP^156^ visit 2
1	530.9	206.2	22.0	26.0	3	559.3	missing	27.0	444.1
2	351.4	200.7	14.9	24.2	7	385.0	171.5	21.3	32.6
4	433.3	200.9	21.6	15.5	8	487.2	2218.1	26.6	1484.6
5	665.6	303.3	40.2	36.6	9	663.2	576.8	17.8	31.2
6	754.2	415.8	17.9	39.5	11	774.5	138.8	46.7	34.2
10	408.3	273.6	43.7	16.7	13	293.4	352.8	18.0	51.6
12	633.1	329.0	31.8	12.4	14	544.9	223.7	17.7	37.0
15	390.7	335.5	19.6	52.2	18	118.4	417.3	12.2	55.4
16	559.7	29.7	61.3	26.2	19	537.6	3871.3	14.6	5000
17	460.9	396.4	18.0	14.1	20	295.0	5933.0	25.2	5000

#### 3.3.2 COMP^156^

At visit 1 the SF COMP^156^ was 29 ​μg/ml [18–40] in TC and 23 ​μg/ml [16–30] in CB. At visit 2, the concentrations were 26 ​μg/ml [17–36] in TC and 1217 ​μg/ml [−245-2679] in CB. The increased concentration of COMP^156^ in CB was statistically significant (p= 0.0069). (Fig. 3c & d) (Table 2).

#### 3.3.3 BGN^262^ and COMP^156^ in serum

For either group, there were no changes in serum concentrations of BGN^262^ or COMP^156^ between the visits (suppl.Fig. 1a-1d, Table 5).

### 3.4 Exploratory results (see suppl.files)

To evaluate the influence of the treatments on joints with low biomarker concentrations (BGN^262^ & COMP^156^) at visit 1 were examined post-intervention.

**Fig. 4:**
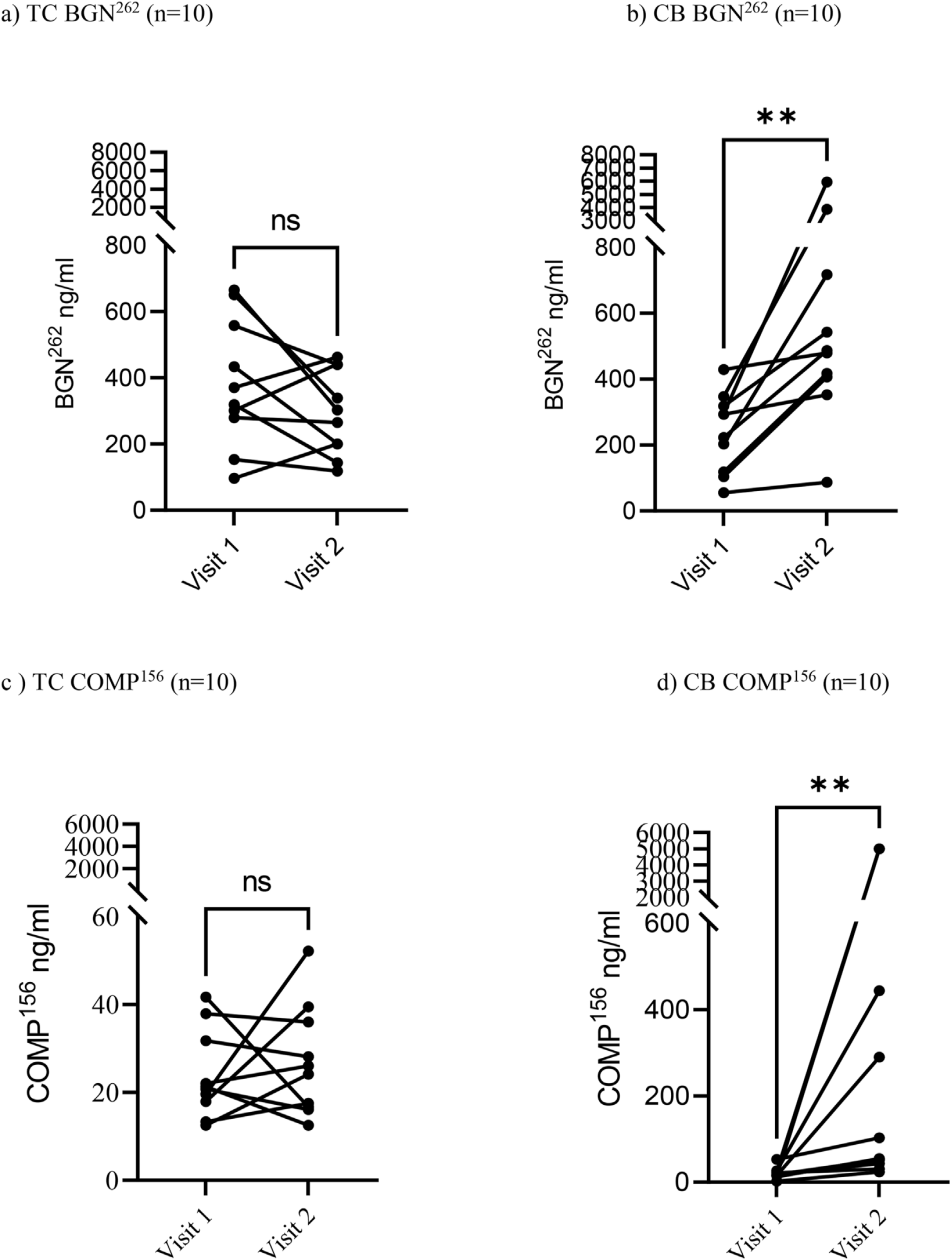
Concentration of BGN^262^ (4a and 4b) and COMP^156^ (4c and 4d) in synovial fluid (from the upper or middle carpal joint with the lowest concentration determined) at visit 1. Both biomarkers for cartilage and bone degradation were increased ( p=0.002 respectively) in the CB group. No changes were observed in TC group

At visit 1, the SF BGN^262^ in TC was 383 ​ng/ml [244–522] and did not change at visit 2 (292 ​ng/ml [201–383]). In CB, the concentrations were 239 ​ng/ml [153–324] and increased significantly to 1330 ​ng/ml [−66 – 2725] (*p=0.002*) (Fig. 4a and b & Suppl. Table 3). The SF COMP^156^ in TC was 24 ​μg/ml [17–31] and did not change at visit 2 (27 ​μg/ml [18–36]). On the other hand, the concentrations in CB were 21 ​μg/ml [11–30] but increased significantly to 1104 ​μg/ml [−368– 2576] (*p=0.002*) at visit 2 (Fig. 4c and d & suppl. Table 4).

### 3.5 Follow-up evaluation by the trainer after 60 days

Two horses in group A were not assessed at visit 4 regarding trotting quality. The trainer couldn't start to train the horse at the time for the interview due to bad winter weather. The horses was therefore given a prolonged rest. The data is treated as missing. At visit 1, the mean trainer-assessed trotting quality was 1.6 in TC and 1.2 in CB, and at follow-up, the mean was 3.5 in TC and 2.2 in CB. The trotting quality improved significantly in TC between visits 1 and 4 but not in CB (*p=0.044*) (suppl.Table 7).

Other parameters such as mood, appetite and fur quality did not differ in the groups between visits 1 and 4.

### 3.6 Monitoring side effects with biomarkers

At visit 2 five of 10 horses in CB showed increased SF COMP^156^ (μg/ml) and BGN^262^ (ng/ml). Additional two horses showed increased SF COMP^156^. The reference value for healthy horses: BGN^262 10^ ≤ 265 ​ng/ml (173±92); COMP^156^ ​≤ ​22.4 ​μg/ml (16.5±5.9) [11] (Table 3).

**Table 3:** Biomarker-assisted monitoring of side effects. Five of 10 horses that received treatment with Celeston® Bifas® showed increased concentrations of COMP^156^ (μg/ml) and BGN^262^ (ng/ml) in SF post-intervention at visit 2. Additional two horses showed increased concentration of BGN^262^ in SF. *CB=Celeston® Bifas®.* As a reference, the value for healthy horses BGN^262^ ≤ 265 ​ng/ml (173±92) [4] and for COMP^156^ ​≤ ​22.4 ​μg/ml (16.5±5.9)^5^.

Horse IDs CB	BGN^262^ ​ng/ml	COMP^156^μg/ml
3	–	444
7	–	289
8	6487	1484
9	6560	5000
11	717	103
19	3871	5000
20	2463	5000

## 4 Discussion

This study aimed to evaluate the efficacy of a new DMOAD combination (TC) with companion diagnostics in relieving joint pain and reducing ECM destruction in both cartilage and SCB against a CB-treated group (positive control) in STBs. Our results are the first to show the efficacy of TC treatment on clinical lameness and reduction in the soluble SF BGN^262^ levels, a novel biomarker reflecting SCB degradation. An increase in SF COMP^156^ (cartilage-derived biomarker) in CB can be addressed as a severe side effect. Both biomarkers meet the companion diagnostics requirements [17].

Predictive soluble biomarkers can be classified according to BIPEDS; Burden of disease (B), Investigative (I), Prognostic (P), Efficacy of intervention (E) Diagnostic (D) and Safety (S) [18]. The reduced BGN^262^ levels in TC indicate impediment of the SCB destruction and are comparable to that of healthy trained horses [10]. BGN^262^ potentially meets the requirement to be an ideal predictive soluble candidate biomarker according to BIPEDs classification; (B) (E) (S) and (D).

The dramatic post-treatment increase of SF COMP^156^ in CB indicates severe cartilage degradation, a serious side effect of corticosteroid therapy. COMP^156^ potentially meets the requirement to be a predictive soluble biomarker according to BIPEDs classification; (B) (S) and (D).

The development of DMOAD drugs follows a new disease subtype classification system (endotypes) considering cellular and molecular signalling pathways [2] Three endotypes; inflammation-driven, bone-driven and cartilage-driven endotypes or six main endotypes; metabolic syndrome-driven, cartilage-driven, ageing-driven, synovitis-driven, mechanical injury-driven and SCB-driven endotypes and their corresponding molecular endotypes have been proposed [5,19].

In this study, the OA-predictive biomarker neoepitopes were found to be capable of defining both clinical phenotypes and their corresponding molecular endotypes (cartilage-driven, bone-driven and mechanical injury-driven) [10,19]. All the study horses had similar demographics (age, sex, breed and athletic profile), targeted OA phenotypes (mild OA in the same joints) and joint pain at inclusion. Additionally, by quantifying soluble BGN^262^ and COMP^156^, we present the possibility of accurately phenotyping the incipient OA in a horse according to the recommeneded/proposed endotypes.

In agreement with the STARD guidelines pertaining to the diagnostic accuracy of a candidate biomarker, we can conclude that the horses in the trial at inclusion could be grouped into endo-types (a) high BGN^262^ (n=19) or (b) high BGN^262^ & COMP^156^ (n=8) (Table 2) with latter reflecting a more severe incipient OA.

The cleavage site within the neo-epitopes (BGN^262^ and COMP^156^) is conserved across the species including humans thus making them ideal biomarker candidates for drug development as they come with a proof-of-concept, safety, surrogate-end-point, and companion diagnostics.

Race horses are more vulnerable in developing OA, especially in high-motion joints such as carpal joint. Therefore, lameness originating from this joint has targeted for treatment. The results clearly indicate that one dose of TC injection was equivalent to 2 doses of CB in subsiding joint pain. At 60 days post-intervention (visit 4 and still blinded) the trotting assessment by the trainer received a better score in TC, suggesting a prolonged treatment benefit.

For several decades, intra-articular treatment with corticosteroids is in use for OA-related pain and remains to be the most used drug in high-motion joints in horses and large joints in humans. Despite their efficacy in releiving joint pain, their repeated use has been reported to have shortcomings. A study with triamcinolone resulted in a greater cartilage volume loss when compared to intra-articular saline injection at two-year follow up [22]. The pros and cons of corticosteroids are unclear [[20], [21], [22]]. However, our study data sides with the negative effect of such joint treatment. A dramatic increase in SF COMP^156^ in CB group indicates cartilage degradation and a severe post-intervention side effect. In an exploratory approach, joints were grouped by low concentrations of biomarkers (BGN^262^ and COMP^156^) at visit 1. This was done to assess the response of such joints to the respective treatments. Both markers showed a significant increase in SF following CB treatment, which was not seen in 10.13039/100006922TC, clearly supporting the dual potential of the biomarkers in rightly diagnosing the affected joint and monitoring the side effects.

Lately, the key signalling molecules investigated for DMOADs targets for human use are transforming growth factor β (TGF- β-cartilage), wingless-related integration site (WNT-cartilage), the transient receptor potential (TRP) channels [5]. The drugs currently in phase II-IV clinical trials are: inhibitors of the Wnt pathways, NF-κB pathway, and Toll-like receptor (TLR) pathway [7] as well as monoclonal antibodies against nerve growth factor (NGF) [23] cathepsin-k inhibitor [24] and recombinant fibroblast FGF-18 ^2^.

The TC drug primarily acts by restoring cell-to-cell signalling which is essentially impaired in OA. Studying basic cellular mechanisms, which is mostly similar in many inflamed cell types (chondrocytes, fibroblast and astrocytes) has been crucial in understanding how different drugs can restore the derailed cellular networks [14,[25], [26], [27], [28]]. Gap junction-coupled cells forming networks in different organs in the body are another focus point [29,30]. Various cell types i.e., astrocytes, osteoblasts, osteocytes, osteoclasts and chondrocytes express connexin 43 (Cx43) and communicate via Ca^2+^ waves [[31], [32], [33], [34]]. Intracellular Ca^2+^ release is controlled by different signalling pathways that can be stimulated by various neurotransmitters, such as ATP, glutamate and 5-HT [[35], [36], [37]].

Local anaesthetic agents, such as bupivacaine and carbocaine®, are widely used clinical agents inducing analgesia by blocking voltage-gated Na^+^ channels when used for neuro-axial blockades. Lower concentrations of bupivacaine unlike clinical doses, evoked Ca^2+^ transients and blocked nerve impulse propagation, in turn may have a pain-relieving effect via targeting G protein-coupled receptors and binding sites on immune cells [[38], [39], [40]].

Lower concentrations of bupivacaine (<10^−8^ ​M) were found to evoke intracellular Ca^2+^ transients that were inositol trisphosphate (IP_3_) receptor-dependent in astrocytes. The concentration-dependent curve for bupivacaine did not follow a Gaussian curve. At higher concentrations, >10^−8^ ​M, bupivacaine blocked the Ca^2+^ release. The absence of such a response at higher concentrations could be due to Na ​^+^ ​channel inhibition [37,41,42]. In addition, we found low concentrations of bupivacaine decreased the inflammation-induced interleukin-1β (IL-1*β*) release in astrocytes [39]. The same results were seen in OA chondrocytes [14]. These results support the anti-inflammatory properties of bupivacaine in low concentrations.

Sildenafil (clinical dose) is a potent and selective PDE-5 inhibitor, which induces cyclic GMP accumulation [43]. This drug at lower concentrations reduces the Ca^2+^ response intensity and induces a more organized actin fiber pattern in inflamed cells [44]. Santillo et al., 2018 [43] studied the dose-response curves for sildenafil and other PDE-5 inhibitor analogues and reported the inhibition dose range to be between 0.1 and 100 ​ng/ml. In our study, the Sildenafil dose was below that dose range, where PDE-5 inhibition is completely abolished. Taken together, the beneficial effect exerted by low doses thus is by balancing Ca^2+^ responses and maintaining the cytoskeleton integrity by direct interaction with Na^+^/K^+^-ATPase as a target.

Glucose, a primary substrate for ATP production (in glycolysis) and for matrix molecule synthesis, such as hyaluronan: crucial in articular cartilage assembly [45]. Inflamed chondrocytes consume more glucose, downregulates the GLUTs thus disrupting the glucose balance [[45], [46], [47]]. Metformin exposure increases cellular glucose consumption and enhances glycolytic flux across the cell membrane [48]. Compared to normal chondrocytes, *in vitro* OA chondrocytes exhibit increased intracellular Ca^2+^ release, associated with TLR4 induction and other inflammatory mediators [27]. Glucose or metformin in combination with an anaesthetic agent and sildenafil restored elevated 5-HT- and ATP-evoked intracellular Ca^2+^ signalling between the cells through gap junctions *in vitro* [13,14]. TLR4 has been identified as a potential drug target for the treatment of inflammatory diseases including OA therefore inhibitors modulating TLR4 signalling in joint tissues have been proposed as DMOADs [49,50]. The prominent downregulation of TLR4 in chondrocytes treated with metformin and bupivacaine further indicates anti-inflammatory properties of this drug combination [14].

We, therefore, aimed to modify the underlying OA pathophysiology by restoring basic cellular parameters to normal physiological state, ie. intracellular Ca^2+^ signalling, increase Na^+^/K^+^-ATPase activity, enhancing glucose uptake and downregulating TLR4. The cartilage and bone cells thereby alleviate the inflammatory mediators expression associated with structural damage in cartilage and SCB.

## 5 Conclusions

This study presents sensitive and specific neo-epitope biomarkers to monitor TC efficacy as well as corticosteroid side effects. TC exhibits DMOAD properties by slowing down OA-associated SCB destruction, revoking lameness and joint pain.

Since validated neo-epitope biomarkers (COMP^156^ & BGN^262^) used in this study are highly conserved across the species makes it possible to put our current study findings into human OA perspective.

## Declarations of interest

ES and AL are stakeholders of SGPTH Life Science AB holding the patent for BGN^262^ and COMP^156^ neo-epitopes.

EH, ES, KA-A and AL are stakeholders of ARTROA AB holding, the patent for pharmaceutical drug combination. The other co-author has no conflicts of interest to declare.

## Fundings

The study was funded by the Grants and Innovation Office at Gothenburg University, Sweden. It had no role in study design, data collection, interpretation or manuscript writing.

## Author contributions

ES and EH designed the study, ES, EH, KAA and AL the overall direction and study implementation. SA, CL, SN, and KB set up and validated the ELISAs, analysed all the samples. ES, SA, CL and EH wrote the manuscript. All authors contributed to and approved the final manuscript.

## References

[1] Vincent T.L. (2020). Of mice and men: converging on a common molecular understanding of osteoarthritis. Lancet Rheumatol.

[2] Makarczyk M.J., Gao Q., He Y., Li Z., Gold M.S., Hochberg M.C. (2021). Current models for development of disease-modifying osteoarthritis drugs. Tissue Eng. C Methods.

[3] Wade C.M., Giulotto E., Sigurdsson S., Zoli M., Gnerre S., Imsland F. (2009). Genome sequence, comparative analysis, and population genetics of the domestic horse. Science.

[4] Ribitsch I., Baptista P.M., Lange-Consiglio A., Melotti L., Patruno M., Jenner F. (2020). Large animal models in regenerative medicine and tissue engineering: to do or not to do. Front. Bioeng. Biotechnol..

[5] Oo W.M. (2022). Prospects of disease-modifying osteoarthritis drugs. Clin. Geriatr. Med..

[6] Cho Y., Jeong S., Kim H., Kang D., Lee J., Kang S.B. (2021). Disease-modifying therapeutic strategies in osteoarthritis: current status and future directions. Exp. Mol. Med..

[7] Kumavat R., Kumar V., Malhotra R., Pandit H., Jones E., Ponchel F. (2021). Biomarkers of joint damage in osteoarthritis: current status and future directions. Mediat. Inflamm..

[8] Cui J., Zhang J. (2022). Cartilage oligomeric matrix protein, diseases, and therapeutic opportunities. Int. J. Mol. Sci..

[9] Miguez P.A. (2020). Evidence of biglycan structure-function in bone homeostasis and aging. Connect. Tissue Res..

[10] Adepu S., Ekman S., Leth J., Johansson U., Lindahl A., Skioldebrand E. (2022). Biglycan neo-epitope (BGN(262)), a novel biomarker for screening early changes in equine osteoarthritic subchondral bone. Osteoarthritis Cartilage.

[11] Skiöldebrand E., Ekman S., Mattsson Hultén L., Svala E., Björkman K., Lindahl A. (2017). Cartilage oligomeric matrix protein neoepitope in the synovial fluid of horses with acute lameness: a new biomarker for the early stages of osteoarthritis. Equine Vet. J..

[12] Ekman S., Lindahl A., Rüetschi U., Jansson A., Björkman K., Abrahamsson-Aurell K. (2019). Effect of circadian rhythm, age, training and acute lameness on serum concentrations of cartilage oligomeric matrix protein (COMP) neo-epitope in horses. Equine Vet. J..

[13] Hansson E., Skiöldebrand E. (2019). Anti-inflammatory effects induced by ultralow concentrations of bupivacaine in combination with ultralow concentrations of sildenafil (Viagra) and vitamin D3 on inflammatory reactive brain astrocytes. PLoS One.

[14] Hansson E., Skiöldebrand E. (2021). Bupivacaine in combination with sildenafil (Viagra) and vitamin D3 have anti-inflammatory effects in osteoarthritic chondrocytes. Curr Res Pharmacol Drug Discov.

[15] Schulz K.F., Altman D.G., Moher D., the C.G. (2010). CONSORT 2010 Statement: updated guidelines for reporting parallel group randomised trials. BMC Med..

[16] Cohen J.F., Korevaar D.A., Altman D.G., Bruns D.E., Gatsonis C.A., Hooft L. (2016). STARD 2015 guidelines for reporting diagnostic accuracy studies: explanation and elaboration. BMJ Open.

[17] Willis J.E., Eyerer F., Walk E.E., Vasalos P., Bradshaw G., Yohe S.L. (2022).

[18] Kraus V.B., Blanco F.J., Englund M., Henrotin Y., Lohmander L.S., Losina E. (2015). OARSI Clinical Trials Recommendations: soluble biomarker assessments in clinical trials in osteoarthritis. Osteoarthritis Cartilage.

[19] Henrotin Y. (2022). Osteoarthritis in year 2021: biochemical markers. Osteoarthritis Cartilage.

[20] Zanotto G.M., Frisbie D.D. (18 June 2021). Current joint therapy usage in equine practice: changes in the last 10 years. Equine Vet. J..

[21] Block J.A. (2022). Are intraarticular glucocorticoids safe in osteoarthritis?. Arthritis Rheumatol..

[22] McAlindon T.E., LaValley M.P., Harvey W.F., Price L.L., Driban J.B., Zhang M. (2017). Effect of intra-articular triamcinolone vs saline on knee cartilage volume and pain in patients with knee osteoarthritis: a randomized clinical trial. JAMA.

[23] Enomoto M., Mantyh P.W., Murrell J., Innes J.F., Lascelles B.D.X. (2019). Anti-nerve growth factor monoclonal antibodies for the control of pain in dogs and cats. Vet. Rec..

[24] Conaghan P.G., Bowes M.A., Kingsbury S.R., Brett A., Guillard G., Rizoska B. (2020). Disease-modifying effects of a novel cathepsin K inhibitor in osteoarthritis: a randomized controlled trial. Ann. Intern. Med..

[25] Hansson E., Werner T., Bjorklund U., Skioldebrand E. (2016). Therapeutic innovation: inflammatory-reactive astrocytes as targets of inflammation. IBRO Rep.

[26] Skioldebrand E., Lundqvist A., Bjorklund U., Sandstedt M., Lindahl A., Hansson E. (2017). Inflammatory activation of human cardiac fibroblasts leads to altered calcium signaling, decreased connexin 43 expression and increased glutamate secretion. Heliyon.

[27] Skiöldebrand E., Thorfve A., Björklund U., Johansson P., Wickelgren R., Lindahl A. (2018). Biochemical alterations in inflammatory reactive chondrocytes: evidence for intercellular network communication. Heliyon.

[28] Hansson E., Björklund U., Skiöldebrand E., Rönnbäck L. (2018). Anti-inflammatory effects induced by pharmaceutical substances on inflammatory active brain astrocytes—promising treatment of neuroinflammation. J. Neuroinflammation.

[29] Hansson E., Skiöldebrand E. (2015). Coupled cell networks are target cells of inflammation, which can spread between different body organs and develop into systemic chronic inflammation. J. Inflamm..

[30] Kettenmann H., Ransom B.R. (1988). Electrical coupling between astrocytes and between oligodendrocytes studied in mammalian cell cultures. Glia.

[31] Giaume C., McCarthy K.D. (1996). Control of gap-junctional communication in astrocytic networks. Trends Neurosci..

[32] Cornell-Bell A.H., Finkbeiner S.M., Cooper M.S., Smith S.J. (1990). Glutamate induces calcium waves in cultured astrocytes: long-range glial signaling. Science.

[33] Donahue H.J., Qu R.W., Genetos D.C. (2017). Joint diseases: from connexins to gap junctions. Nat. Rev. Rheumatol..

[34] Li Z., Huang Z., Bai L. (2021). Cell interplay in osteoarthritis. Front. Cell Dev. Biol..

[35] Blomstrand F., Khatibi S., Muyderman H., Hansson E., Olsson T., Rönnbäck L. (1999). 5-Hydroxytryptamine and glutamate modulate velocity and extent of intercellular calcium signalling in hippocampal astroglial cells in primary cultures. Neuroscience.

[36] Muyderman H., Angehagen M., Sandberg M., Björklund U., Olsson T., Hansson E. (2001). Alpha 1-adrenergic modulation of metabotropic glutamate receptor-induced calcium oscillations and glutamate release in astrocytes. J. Biol. Chem..

[37] Hansson E., Rönnbäck L. (2003). Glial neuronal signaling in the central nervous system. Faseb. J..

[38] Toda S., Sakai A., Ikeda Y., Sakamoto A., Suzuki H. (2011). A local anesthetic, ropivacaine, suppresses activated microglia via a nerve growth factor-dependent mechanism and astrocytes via a nerve growth factor-independent mechanism in neuropathic pain. Mol. Pain.

[39] Block L., Jörneberg P., Björklund U., Westerlund A., Biber B., Hansson E. (2013). Ultralow concentrations of bupivacaine exert anti-inflammatory effects on inflammation-reactive astrocytes. Eur. J. Neurosci..

[40] Amir R., Argoff C.E., Bennett G.J., Cummins T.R., Durieux M.E., Gerner P. (2006). The role of sodium channels in chronic inflammatory and neuropathic pain. J. Pain.

[41] Hansson E. (2010). Long-term pain, neuroinflammation and glial activation. Scand J Pain.

[42] Forshammar J., Block L., Lundborg C., Biber B., Hansson E. (2011). Naloxone and ouabain in ultralow concentrations restore Na+/K+-ATPase and cytoskeleton in lipopolysaccharide-treated astrocytes. J. Biol. Chem..

[43] Santillo M.F., Mapa M.S.T. (18 Feb 2018). Phosphodiesterase (PDE5) inhibition assay for rapid detection of erectile dysfunction drugs and analogs in sexual enhancement products. Drug Test. Anal..

[44] de Santana Nunes A.K., Rapôso C., Björklund U., da Cruz-Höfling M.A., Peixoto C.A., Hansson E. (2016). Sildenafil (Viagra(®)) prevents and restores LPS-induced inflammation in astrocytes. Neurosci. Lett..

[45] Rotter Sopasakis V., Wickelgren R., Sukonina V., Brantsing C., Svala E., Hansson E. (2019). Elevated glucose levels preserve glucose uptake, hyaluronan production, and low glutamate release following interleukin-1β stimulation of differentiated chondrocytes. Cartilage.

[46] Li K., Ji X., Seeley R., Lee W.C., Shi Y., Song F. (2022). Impaired glucose metabolism underlies articular cartilage degeneration in osteoarthritis. Faseb. J..

[47] Mobasheri A., Vannucci S.J., Bondy C.A., Carter S.D., Innes J.F., Arteaga M.F. (2002). Glucose transport and metabolism in chondrocytes: a key to understanding chondrogenesis, skeletal development and cartilage degradation in osteoarthritis. Histol. Histopathol..

[48] Westhaus A., Blumrich E.M., Dringen R. (2017). The antidiabetic drug metformin stimulates glycolytic lactate production in cultured primary rat astrocytes. Neurochem. Res..

[49] Gómez R., Villalvilla A., Largo R., Gualillo O., Herrero-Beaumont G. (2015). TLR4 signalling in osteoarthritis--finding targets for candidate DMOADs. Nat. Rev. Rheumatol..

[50] Ain Q.U., Batool M., Choi S. (2020). TLR4-Targeting therapeutics: structural basis and computer-aided drug discovery approaches. Molecules.

