# Supplementary material for: A randomized, triple-blinded controlled clinical study with a novel disease-modifying drug combination in equine lameness-associated osteoarthritis
Source: Osteoarthr Cartil Open. 2023 Jun 16;5(3):100381. doi: 10.1016/j.ocarto.2023.100381 (PMC10320210; doi:10.1016/j.ocarto.2023.100381)
Supplement: Multimedia component 1 [file mmc1.pdf]

Supplementary data

Material and Methods

Objectively inspection of gait asymmetry with Lameness locator®

For the initial lameness and after intra-articular anaesthesia the gait asymmetry was monitored with a multi-sensor inertial gait analysis system (Lameness Locator® 2017, Equinosis). It detects and quantifies movement asymmetry using small wireless inertial sensors mounted on the horse, joined via a long-range wireless connection to a hand-held tablet computer. Four sensors were placed at the head at the poll, the withers, the right pastern and one at the hip. The system samples motion about 10 times faster and more sensitively than the human eye. The software utilizes special algorithms developed over years to measure and analyze lameness and filter insignificant movement. The Q score parameter is a metric for the forelimb evaluation, a quantification of asymmetry, this being a summary of the measurements provided by the limb, the time and the amplitude of the asymmetry, regardless of whether the asymmetry is above or below the defined thresholds (www.equinosis.com). The cut-off value for a Qscore value for a sound horse is 6 mm[1].

Visual inspection of lameness after a flexion test

The veterinarian performed a flexion test by holding the horse's forelimb in a flexed position for 60 s, followed by trotting immediately in a straight line on a hard surface[2]. As the horse trots, the veterinarian watches for signs of pain, weight shifting or irregular movement and the lameness was graded on a scale of 0–5[3], (0= sound, 5= non-weight bearing) (Table 1).

Sampling of SF and serum for biomarkers

The samples were centrifuged at 5700 g for 15 min and supernatant aliquots were frozen initially at -20°C for one day before being transferred to the -80°C freezer (Sahlgrenska University Hospital).

Blood samples were collected and stored at room temperature (RT) for 90-120 min and then centrifuged at 5700 g for 10 min. The serum was separated and stored first at -20°C for one day and then transferred to -80°C until analysis (Sahlgrenska University Hospital).

All samples were marked with pre-printed labels and recorded with an ID number, joint compartment (SF), date and name of the horse.

BGN^{262} ELISA

The ELISA for BGN^{262} was performed as follows. Nunc MaxiSorp™ Clear Flat-Bottom 96-Well Plates (Invitrogen) were coated with BGN^{262} peptide (1 ug/mL, 100µl/well, Gen-Script Biotech, The Netherlands), diluted in 100 mM carbonate buffer (pH 9.6) and incubated overnight at 4°C. The calibration standard was prepared from the 1 mg/mL stock of the BGN^{262} peptide. The highest standard point was set at 2 mg/mL by diluting in Effect Diluent (Kementech, Denmark) and thereafter, an 11-step 1:2 serial dilution in Effect Diluent buffer giving a range of 0 (the 11th with no peptide) to 2 µg/mL standard curve. The samples and standards were diluted in Effect Diluent buffer, Kementech, Denmark). The sample dilution used was 1:4 for synovial fluid and 1:20 for serum, as determined earlier [4]. The primary monoclonal antibody against BGN^{262} (0.681 mg/mL, lot: U8229DL260-6, GenScript) was diluted in Effect Diluent buffer to a concentration of 30 ng/mL. The calibration standards and samples (100 µl /well, in duplicate) were added to Thermo Scientific™ Sterilin ™ Clear Microtiter™ Plates (Fisher Scientific). The primary antibody (100 µl /well) was added to each standard as well as samples and pre-incubated overnight in a humid chamber within a rotation incubator (39 rpm) at 37°C. After 17 h, the previously coated ELISA plate was washed 4 times in the wash buffer (10 mM PBS with 0,05 % Tween, pH 7,4) using Tecan Hydro wash and thereafter blocked with Synthetic Blocker (Kementech, Denmark) for 30 min at 37ºC. After blocking, the pre-incubated standards and samples (100 µl /well) was transferred to the

ELISA plate and incubated for 1 h at room temperature on an ELISA plate shaker set at 600 rpm. After incubation, the ELISA plate with the primary antibody, standard, and samples was washed four times with the wash buffer. The secondary polyclonal (Goat Anti-Rabbit IgG H&L [HRP][Ab97051]) was diluted 1:50,000 in 10 mM PBS with 0, 05 % Tween and 0.1% BSA, pH 7, 4 and was added to the standard and sample wells (100 µl /well) in the ELISA plate, incubated in the dark for 30 min on an ELISA shaker set at 600 rpm. Thereafter, the ELISA plate was washed eight times with wash buffer. Next, the TMB (100 µl L/well) was added and incubated in the dark at RT, and the reactions were stopped after 12 minutes with 0.18 M H2SO4 (100 µl /well). The absorbance was measured at 450 nm using SPARK multifunctional plate reader with the Magellan software (Tecan).

COMP^{156} ELISA

The ELISA for COMP^{156} was performed as follows[5]. NUNC ELISA plates were coated with 4.0 ug/mL peptide (sequence SGPTHEGVC) diluted in 0.1 mol/L carbonate buffer, pH 9.6 and incubated at 4°C overnight. A 1:2 serial dilution of 5.0 µg/mL peptide (sequence SGPTHEGVGMA) in 10 mmol/L phosphate-buffered saline (PBS) with 0.6% BSA and 0.8% SDS was used as a calibration curve (range = 5–0.078 µg/mL). SF samples were diluted 1:20 in PBS with 0.84% SDS. Duplicates of standard and synovial fluid were overnight-incubated in 96-well Thermo Scientific™ Sterilin ™ Clear Microtiter™ Plates (Fisher Scientific) at 25°C. On the second day, primary polyclonal anti-COMP antibody (diluted 1:2000 in PBS with 1% BSA and 4% Triton-X-100) was added to the Sterilin plates and the plates were incubated for 1 h 20 min at 25°C. The Coated ELISA plates were washed and blocked (PBS with 1% BSA and 0.1% Tween) for 1 h at 25°C. A total of 100 µl was transferred from the Sterilin plates to the ELISA plates and incubated for 1 h at 25°C. After incubation, the ELISA plates were washed 4 times and the secondary antibody (Goat Anti-Rabbit IgG H&L

[HRP][Ab97051]) was diluted 1:20,000 in PBS with 1% BSA and 0.1% Tween and 100 uL/well was added. The plates were incubated for 1 h at 25°C and then washed 6 times and incubated with the substrate for approximately 8 min at 25°C. The reaction was stopped with 100 µmol/L H2SO4 and the absorbance was measured at 450 nm. The linearity was tested via the serial dilution (1:5–1:120) of 2 synovial fluid samples

The specificity of the primary antibodies against the neo-epitopes was evaluated using overlapping peptides, as coating peptides and as antigens in the preincubation, where serial dilutions were made similar to the calibration standard. The intra-assay precision was assessed using a control serum (commercially purchased from Håtunalab AB, Håtunaholm, Sweden) in six replicates. The inter-assay was also examined for the control serum as six replicates in a total of three assays on different occasions. All samples were run in duplicates and the concentrations were normalised to the control serum that was run in each plate.

In addition to the standard curve, an aliquoted serum sample was run on all plates for normalisation and comparison of results between plates. The normalised values were used in the statistical evaluation.

Interview questionaire

visit 1

1. How do you rate the horse´s trot?

2. How is the horse´s appetite?

3. How is the horse´s mood?

4. How is the quality of the horse´s coat?

The answers were graded 1-4; 1 = bad, 2 = normal, 3 = good, 4 = very good. The responses were recorded in the journal.

visit 4

At follow-up 60 days post-treatment the trainers (professionals and amateurs) were interviewed on the telephone. The same questions as at visit 1 were asked:

1. How do you rate the horse´s trot?

2. How is the horse´s appetite?

3. How is the horse´s mood?

4. How is the quality of the horse´s coat?

The answers were graded 1-4; 1 = bad, 2 = normal, 3 = better, 4 = much better. The responses were recorded in the journal.

Results

BGN^{262} and COMP^{156} in serum

At visit 1, BGN^{262} concentration in serum was 1526 ng/ml [1392-1661] for the TC horses and 1477 ng/ml [1291-1663] for the CB group. The serum COMP^{156} concentrations were 6 µg/ml [4 -7] in TC horses and 6 µg/ml [4 -8] in CB horses.

For either group, there were no changes in serum concentrations of BGN^{262} or COMP^{156} between the visits (Figure 1a-1d, Table 5).

Lameness Locator (Q-score)

At visit 1, the initial lameness evaluated with Lameness Locator® (Q-score) was 13.6 ± 5.7 (TC horses) and 10.7±9.2 (CB horses). At visit 2, the Q-score values dropped to 9 ± 7 for TC horses and 8.9±12 for CB horses and at visit 3 the values were 8±7.7 and CB 5±5 for TC and CB horses, respectively. According to a cut-off value of 6 mm for a healthy horse, the initial lameness for all horses in the CB group was considered non-lame at visit 3 (Table 6).

Safety

No clinical symptoms of adverse effects were observed for horses post-treatment either TC or CB group and the blood sample parameters did not change over time (Supplementary data blood sample-safety report).

Figures and Tables

Figure 1
Concentration of BGN ^{262} (ng/ml) in serum at visits 1,2 and 3 for
1a) TC and 1b) CB groups. No significant differences were found for either by treatment or by visit.
Concentration of COMP^{156} (µg/ml) in serum at visits 1, 2 and 3 for 1c) TC (n=10) and 1d) CB (n=10). No significant differences was found for neither treatment nor visit.

TC= treatment combination, CB=Celeston® Bifas®.

1a) TC

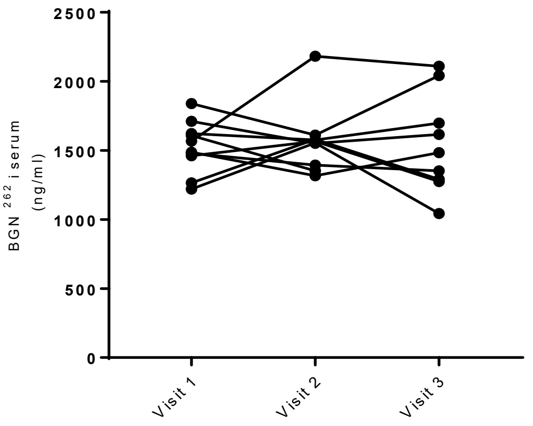

1b) CB

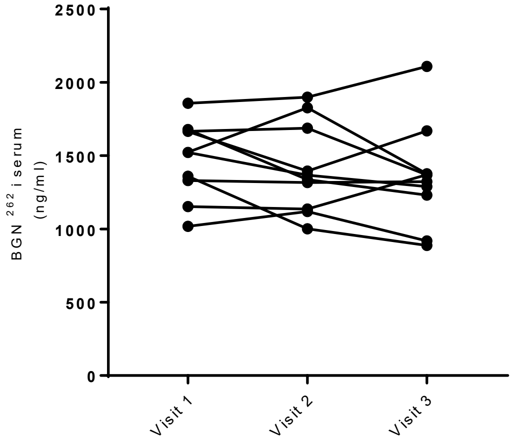

1c) TC

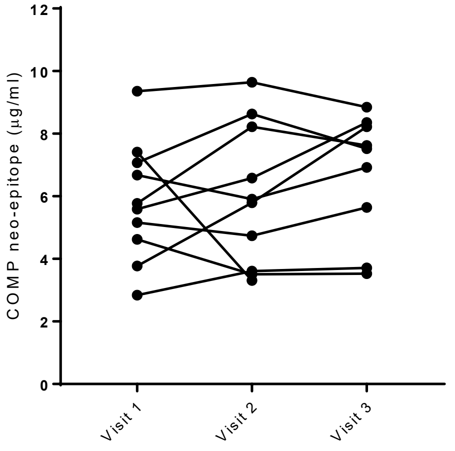

1d) CB

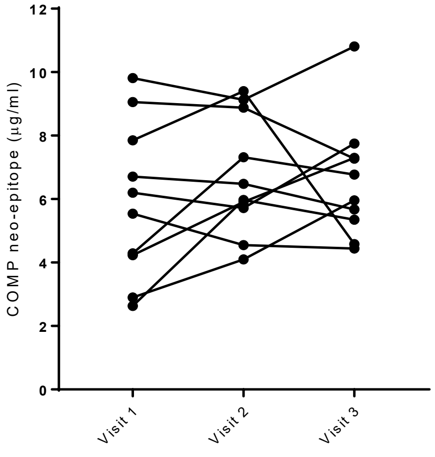

Table 1: A grading system of lameness described by Dr Ross[3] and Dr Dyson[6]

Lameness grades from 0–5 are based on observation of the horse at a trot in hand, in a straight line, on a firm or hard surface.
0= Sound
1=Mild lameness observed while the horse is trotted in a straight line.When the lame forelimb strikes, a subtle head nod is observed; whenthe lame hindlimb strikes, a subtle pelvic hike occurs. The head nodand pelvic hike may be inconsistent at times.
2= Obvious lameness is observed. The head nod and pelvic hike are seenconsistently and excursion is several cm.
3= Pronounced head nod and pelvic hike of several cm are noted. If thehorse has unilateral singular hindlimb lameness, a head and neck nodis seen when the diagonal forelimb strikes the ground (mimickingipsilateral forelimb lameness).
4=Severe lameness with extreme head nod and pelvic hike is present. The horse can still be trotted, however.
5=The horse does not bear weight on the limb. If trotted, the horse carries the limb (horses that are nonweightbearing at the walk or whilestanding should not be trotted).

Table 2. Flexion test reaction (graded 0-5) at visit 1, 2 and 3.

TC= treatment combination, CB=Celeston® Bifas®.

Horse IDs TC	Visit 1	Visit 2	Visit 3	Horse IDs CB	Visit 1	Visit 2	Visit 3
1	2	0	0	7	1.0	1.0	0
5	1	0	0	8	1.0	1.0	0
10	1.5	0	0	9	2.0	1.0	0
16	1.5	0	0	11	1.5	1.0	0
17	1.5	0	0	13	2.0	1.0	0
2	1	0.5	0	14	1.5	1.0	0
6	2	1.5	0	18	1.5	0.5	0
12	2	1.0	0	19	1.5	0.5	0
4	1.5	1.0	1.0	3	1.0	0	0.5
15	1.0	1.0	0.5	20	1.0	1.0	0.5

Table 3. Carpal joints (upper or middle joint compartment) with a low concentration of BGN[262] (ng/ml) in SF at visit 1 in the TC and CB groups and the comparison with visit 2. The values represent mean of the assay duplicates.

TC= treatment combination, CB=Celeston® Bifas®.

Horse IDs TC	BGN[262] Visit 1	BGN[262] Visit 2	Horse IDs CB	BGN[262] Visit 1	BGN[262] Visit 2
1	319.3	143.6	3	429.0	479.4
2	96.7	200.7	7	55.8	87.3
4	433.3	201.0	8	103.7	406.8
5	665.7	303.3	9	318.3	543.0
6	650.7	339.5	11	203.2	717.8
10	153.8	118.4	13	293.4	352.8
12	558.4	440.0	14	223.1	487.1
15	301.6	441.5	18	118.4	417.3
16	280.0	265.0	19	347.5	3871.3
17	370.5	463.0	20	295.0	5933.0

Table 4. Carpal joints (upper or middle joint compartment) with low concentration of COMP[156] (µg/ml) in SF at visit 1 in the TC and CB groups and the comparison with visit 2. The values represent mean of the assay duplicates.

TC= treatment combination, CB=Celeston® Bifas®.

Horse IDs TC	COMP[156] Visit 1	COMP[156] Visit 2	Horse IDs CB	COMP[156] Visit 1	COMP[156] Visit 2
1	22.0	26.0	3	27.0	444.1
2	12.5	24.1	7	15.8	290.0
4	21.3	12.5	8	21.8	30.6
5	37.9	36.0	9	16.3	43.8
6	18.0	39.5	11	53.0	103.2
10	31.8	28.2	13	18.0	51.6
12	20.8	16.1	14	2.4	23.7
15	19.6	52.2	18	12.2	55.4
16	41.7	16.7	19	14.6	5000
17	13.4	17.5	20	25.1	5000

Table 5. Concentration of BGN ^{262} (ng/ml) and COMP^{156} (µg/ml) in serum at visits 1, 2 and 3 for the TC and CB groups. Mean [95% CI].

TC= treatment combination, CB=Celeston® Bifas®.

BGN^{262}(ng/ml)	Visit 1	Visit 2	Visit 3
TC	1526[1392-1661]	1569[1397-1740]	1546[1271-1821]
CB	1477[1291-1663]	1409[1191-1626]	1354[1105-1604]
COMP^{156} (µg/ml)	Visit 1	Visit 2	Visit 3
TC	6[4-7]	6[4-8]	7[5-8]
CB	6[4-8]	7[5-8]	7[5-8]

Table 6. Descriptive statistics of the Q-score for visits 1, 2 and 3 for the TC and CB groups.

TC= treatment combination, CB=Celeston® Bifas®.

	TC	CB
Q-score visit 1		
min:	0	0
mean (sd)	13.61 (5.74)	10.67 (9.27)
median (IQR)	14.35 (12.30, 16.02)	12.40 (1.18, 16.38)
max:	21.4	27.5
>0, n (%)	9 (90)	7 (70)
Q-score visit 2		
min:	0	0
mean (sd)	9.03 (6.96)	8.92 (12.01)
median (IQR)	10.15 (2.05, 13.47)	2.00 (0.00, 16.95)
max:	17.9	29.4
>0, n (%)	7 (70)	5 (50)
Q-score visit 3		
min:	0	0
mean (sd)	8.05 (7.72)	5.08 (5.09)
median (IQR)	8.75 (0.00, 13.92)	5.35 (0.00, 8.30)
max:	19.9	13.4
>0, n (%)	6 (60)	6 (60)

Table 7. Trotting score for TC and CB groups at visit 1a) and visit 4 b) according to the interview of the trainers. There was no significant difference between the treatment groups, (p = 0.650) at visit 1. However, horses in the TC group had a significantly better trotting score between visits 1 and 4 (p = *0.044*). Trotting quality score: 1= bad, 2= normal, 3=good and 4= better

TC= treatment combination, CB=Celeston® Bifas®.

a)

Treatment vs Trotting score at visit 1					
Trotting score	1	2	3	4	Total
TC	5	4	1	0	10
CB	7	3	0	0	10
Total	12	7	1	0	20

b)

Treatment vs Trotting score at visit 4					
Trotting score	1	2	3	4	Total
TC	0	0	4	4	8
CB	3	3	3	1	10
Total	3	3	7	5	18

References:

1 Keegan, K. G. *et al.* Assessment of repeatability of a wireless, inertial sensor-based lameness evaluation system for horses. *Am J Vet Res* **72**, 1156-1163 (2011). https://doi.org:10.2460/ajvr.72.9.1156

2 Verschooten, F. & Verbeeck, J. Flexion test of the metacarpophalangeal and interphalangeal joints and flexion angle of the metacarpophalangeal joint in sound horses. *Equine veterinary journal* **29 1**, 50-54 (1997).

3 Ross, M. W. in *Diagnosis and Management of Lameness in the Horse (Second Edition)* (eds Mike W. Ross & Sue J. Dyson) 64-80 (W.B. Saunders, 2011).

4 Adepu, S. *et al.* Biglycan neo-epitope (BGN(262)), a novel biomarker for screening early changes in equine osteoarthritic subchondral bone. *Osteoarthritis Cartilage* (2022). https://doi.org:10.1016/j.joca.2022.07.005

5 Skiöldebrand, E. *et al.* Cartilage oligomeric matrix protein neoepitope in the synovial fluid of horses with acute lameness: A new biomarker for the early stages of osteoarthritis. *Equine Vet J* **49**, 662-667 (2017). https://doi.org:10.1111/evj.12666

6 Dyson, S. Can lameness be graded reliably? *Equine Vet J* **43**, 379-382 (2011). https://doi.org:10.1111/j.2042-3306.2011.00391.x

		Group Treatment combination				Group Celeston Bifas			
**Variabel**	**Visit**	**Mean**	**sd**	**Number outside reference**	**Number poitive**	**Mean**	**sd**	**Number outside reference**	**Number positive**
Albumin	1	33,06	1,81	0		33,27	1,49	0	
Albumin	2	34,11	1,69	0		33,29	2,03	0	
Albumin	3	34,36	1,70	0		32,37	1,77	0	
Anisocytosis	1			0	0			0	0
Anisocytosis	2			0	0			0	0
Anisocytosis	3			0	0			0	0
AP	1	83,50	21,24	0		80,80	20,75	0	
AP	2	84,00	20,92	0		80,89	29,62	0	
AP	3	84,30	14,62	0		81,90	29,09	0	
AST.(GOT)	1	157,07	27,18	0		160,39	39,93	1	
AST.(GOT)	2	168,39	47,34	1		142,89	32,54	0	
AST.(GOT)	3	168,65	43,59	1		146,72	39,90	0	
Band.neutrophiles	1	-	-	0		-	-	0	
Band.neutrophiles	2	-	-	0		-	-	0	
Band.neutrophiles	3	-	-	0		-	-	0	
Band.neutrophiles_D	1	-	-	0		-	-	0	
Band.neutrophiles_D	2	-	-	0		-	-	0	
Band.neutrophiles_D	3	-	-	0		-	-	0	
Basophiles	1	0,33	0,50	0		0,40	0,52	0	
Basophiles	2	0,70	1,06	2		0,50	0,53	0	
Basophiles	3	0,20	0,42	0		0,10	0,32	0	
Basophiles_D	1	0,03	0,05	0		0,04	0,05	0	
Basophiles_D	2	0,07	0,11	2		0,03	0,05	0	

Basophiles_D	3	0,02	0,04	0	0,01	0,03	0
Bilirubin.total	1	36,71	10,56	1	36,74	8,39	0
Bilirubin.total	2	37,32	13,47	1	35,00	11,84	1
Bilirubin.total	3	39,67	15,79	1	34,22	13,98	1
Calcium	1	2,97	0,09	0	2,92	0,13	0
Calcium	2	2,91	0,11	0	2,92	0,16	0
Calcium	3	3,11	0,27	3	2,95	0,20	0
CK	1	86,30	14,19	0	95,30	20,71	0
CK	2	96,50	24,79	1	90,60	15,44	0
CK	3	109,30	36,64	1	99,70	21,74	1
Copper	1	13,70	2,43	0	12,40	1,39	0
Copper	2	12,90	1,95	0	11,52	2,00	0
Copper	3	12,02	1,92	0	12,63	3,03	0
Eosinophiles	1	2,56	1,59	3	2,70	1,49	3
Eosinophiles	2	2,70	1,06	3	2,50	1,72	3
Eosinophiles	3	3,20	2,25	3	2,30	1,34	2
Eosinophiles_D	1	0,23	0,18	4	0,21	0,14	4
Eosinophiles_D	2	0,26	0,11	4	0,21	0,14	4
Eosinophiles_D	3	0,28	0,20	3	0,20	0,12	3
Erythrocytes	1	8,92	0,98	0	8,67	0,55	0
Erythrocytes	2	9,22	1,22	0	8,65	1,23	0
Erythrocytes	3	8,83	0,97	0	8,09	0,91	0
g-GT	1	9,58	4,12	0	9,81	6,71	1
g-GT	2	8,22	3,13	0	9,19	5,81	0
g-GT	3	8,10	2,69	0	8,56	5,15	0
GLDH	1	0,89	0,31	0	1,45	1,66	0
GLDH	2	0,99	0,48	0	1,51	1,31	0
GLDH	3	1,09	0,58	0	1,64	2,41	1
Globulins	1	26,06	1,72	0	27,10	3,25	0

Globulins	2	26,44	2,75	0		26,98	2,62	0	
Globulins	3	26,96	2,08	0		24,43	7,93	0	
Glukos	1	5,16	0,64	6		4,88	0,46	4	
Glukos	2	4,85	0,39	4		4,76	0,22	2	
Glukos	3	4,97	0,62	3		4,99	0,37	6	
Hematocrit	1	0,38	0,03	0		0,38	0,03	0	
Hematocrit	2	0,39	0,02	0		0,38	0,04	0	
Hematocrit	3	0,37	0,02	0		0,35	0,03	0	
Hemoglobin	1	140,11	10,60	0		140,40	10,89	0	
Hemoglobin	2	145,20	10,04	0		138,80	17,03	1	
Hemoglobin	3	141,40	12,69	0		129,00	11,34	0	
Hypochromasia	1			0	0			0	0
Hypochromasia	2			0	0			0	0
Hypochromasia	3			0	0			0	0
Iron	1	22,63	5,58	1		27,29	8,64	2	
Iron	2	23,87	5,92	1		27,08	6,64	0	
Iron	3	25,01	8,78	3		24,48	7,36	2	
Kolesterol	1	1,73	0,20	6		1,96	0,35	3	
Kolesterol	2	1,86	0,16	5		1,94	0,28	5	
Kolesterol	3	1,88	0,14	5		1,86	0,27	5	
Kreatinine	1	115,00	19,15	0		114,10	17,01	0	
Kreatinine	2	118,80	21,20	0		111,00	12,41	0	
Kreatinine	3	117,80	24,14	0		112,20	15,89	0	
LDH	1	364,24	48,84	3		404,54	95,37	5	
LDH	2	383,69	45,54	3		382,51	106,59	3	
LDH	3	408,08	59,49	6		394,72	89,91	5	
Leucocytes	1	8,88	1,97	4		7,97	1,03	1	
Leucocytes	2	8,83	0,83	1		7,91	2,26	1	
Leucocytes	3	8,91	0,79	0		8,09	2,36	2	

Lymfocytes	1	33,11	5,13	0	33,20	7,22	0
Lymfocytes	2	37,40	8,60	2	35,20	6,44	1
Lymfocytes	3	34,90	4,84	0	33,00	9,45	2
Lymfocytes_D	1	2,91	0,64	1	2,63	0,55	0
Lymfocytes_D	2	3,26	0,63	2	2,95	1,35	1
Lymfocytes_D	3	3,09	0,36	0	2,64	1,01	3
Magnesium	1	0,66	0,05	0	0,68	0,08	0
Magnesium	2	0,70	0,05	0	0,70	0,05	0
Magnesium	3	0,73	0,08	0	0,68	0,06	0
Monocytes	1	4,67	0,87	6	4,70	1,16	5
Monocytes	2	3,90	1,29	4	4,90	1,45	6
Monocytes	3	4,40	1,07	6	5,10	2,02	5
Monocytes_D	1	0,40	0,12	4	0,36	0,10	5
Monocytes_D	2	0,36	0,12	6	0,37	0,08	5
Monocytes_D	3	0,39	0,12	6	0,40	0,15	6
Neutrophiles	1	59,33	5,12	0	59,00	8,22	2
Neutrophiles	2	55,30	8,87	2	55,90	5,63	0
Neutrophiles	3	57,30	6,38	0	59,50	10,59	3
Neutrophiles_D	1	5,31	1,38	1	4,72	1,00	0
Neutrophiles_D	2	4,92	1,04	0	4,38	1,06	0
Neutrophiles_D	3	5,12	0,87	0	4,84	1,74	3
Phosphate	1	1,13	0,21	0	1,05	0,20	0
Phosphate	2	1,11	0,17	1	0,94	0,21	1
Phosphate	3	1,10	0,13	0	1,05	0,20	0
Potassium	1	3,70	0,19	0	3,99	0,38	1
Potassium	2	3,90	0,32	0	3,90	0,49	0
Potassium	3	3,96	0,44	0	3,94	0,34	0
Selenium	1	176,95	32,34	3	155,76	44,30	3
Selenium	2	156,43	25,29	1	144,92	45,30	3

Selenium	3	143,94	18,61	0	131,59	39,23	1
Sodium	1	137,40	1,35	0	136,60	1,43	0
Sodium	2	137,80	2,35	0	137,10	2,69	0
Sodium	3	136,10	2,13	0	138,10	1,91	0
Thrombocytes	1	128,56	17,08	0	135,90	25,56	0
Thrombocytes	2	132,60	14,68	0	139,20	31,39	0
Thrombocytes	3	129,10	19,23	0	140,90	41,17	1
Total.protein	1	59,12	1,82	0	60,37	3,55	0
Total.protein	2	60,55	3,35	0	60,27	2,96	0
Total.protein	3	61,36	2,09	0	58,60	2,76	1
Triglycerides	1	0,26	0,10	0	0,25	0,08	0
Triglycerides	2	0,24	0,05	0	0,24	0,07	0
Triglycerides	3	0,26	0,08	0	0,23	0,08	0
Urea	1	5,42	0,76	1	5,25	0,80	1
Urea	2	4,48	0,85	0	5,19	0,94	0
Urea	3	5,32	1,31	2	5,20	0,93	0
Zinc	1	8,26	1,25	7	9,54	2,83	4
Zinc	2	8,88	1,93	7	9,21	1,91	4
Zinc	3	8,80	1,54	6	9,99	3,74	6

DocuSign Envelope ID: 06A04576-8AB2-4997-AFCE-75ABF2223EF0

Statistikkonsulterna
JOSTAT & MR SAMPLE AB

Statistical analyzes and results of primary and secondary outcomes

Study title: Intra articular treatment with a new drug combination in comparison with Celeston Bifas in horses with lameness associated with osteoarthritis (OA-252)

**Date:**	**Authors:**
2022-06-22	Magnus Pettersson
**Status**:	
Final	Rebecca Bylund

DocuSigned by:
Magnus Pettersson
2B47B83A416F4A2...
DocuSigned by:
Rebecca Bylund
69BAF243696D455...

Summary

This report contains results for primary and secondary analyzes in accordance with SAP for the study "*Intra-articular treatment with a new drug combination in comparison with Celeston bifas in horses with lameness associated with Osteoarthritis*". This is the first of two results reports, where result report part 2 contains results for the exploratory analyzes.

DocuSign Envelope ID: 06A04576-8AB2-4997-AFCE-75ABF2223EF0

TABLE OF CONTENTS

Summary...1

1. Assumptions and statistical methods 3

1.1 Assessment of distribution 3

1.2 Blinding 3

1.3 Handling extreme values 3

1.4 Analysis software 3

2. Primary results 4

2.1 Concentration of BGN 262 in the carpal joint (upper or middle joint compartment) with the highest value at visit 1 4

2.2 Concentration of COMP 1 in the carpal joint (upper or middle joint compartment) with the highest value at visit 1 6

3. Secondary results 7

3.1. Concentration of BGN 262 in synovial fluid in the middle joint compartment of the carpal joint 7

3.2 Concentration of COMP 1 in synovial fluid the middle joint compartment of the carpal joint 8

3.3 Comparison of the number of non-lame (lameness = 0 after flexion test) horses at visit 2 9

3.4 Comparison of the number of non-lame horses (lameness = 0 after flexion test) at visit 3 10

3.5 Comparison of the number of non-lame (after flexion test) horses at both visits 2 and 3 10

3.6 Results for blood samples analysis 12

References 13

Appendix A. Normal Distribution Test 14

Appendix B. Demographic data and Baseline values 16

Appendix C. Figures for concentration of biomarkers 18

DS FB DS MP

DocuSign Envelope ID: 06A04576-8AB2-4997-AFCE-75ABF2223EF0

1. ASSUMPTIONS AND STATISTICAL METHODS

1.1 Assessment of distribution

Assessment of the normal distribution assumption for the concentration of biomarkers (BGN 262 and COMP 1 in synovial fluid) have been made for both non-logarithmic and logarithmic values. Tests figures, and assessment are presented in Appendix A of the report. The normal distribution assumption has been evaluated in two ways:

1) By analyzing the distribution of the error terms for linear mixed models
2) By analyzing the distribution for differences in concentration between visits 1 and 2

Shapiro Wilk's test has been made for both cases and the results are presented in Appendix A. In short, the analyzes show that data deviate from normal distribution, which means that a non-parametric method has primarily been chosen to perform the analyzes on concentration of the biomarkers in synovial fluid.

The statistical methods used to replace linear mixed models are Wilcoxon signed rank test and Wilcoxon rank sum test. For these methods, the same results are obtained for logarithmic and non-logarithmic values as the tests are rank based. Estimates and confidence intervals for original (non-logarithmic) values are also presented.

For tests of two proportions, the Z-test has been replaced with Fisher's exact test, because in all cases there was some cells with too few values (< 5).

1.2 Blinding

The data has been analyzed blindly, and the statisticians have not had access to the code for the treatment arm. The code was broken after the primary and secondary analyzes were completed.

1.3 Handling extreme values

For the concentration of the biomarkers BGN 262 and COMP 1 at visit 2, there are values that appear extreme in relation to the others. There are 4 horses where the concentration of the biomarker in question has increased very much between visits 1 and 2. For BGN 262 it appears for 3 horses and for COMP 1 it appears for 3 horses. Of the total of 4 horses, 2 horses show extreme values for both biomarkers. Furthermore, 2 horses show only extreme values for one of the 2 biomarkers.

Appendix C includes figures showing the difference in concentration between visit 2 and visit 1 where this is illustrated.

These values are one reason why the normal distribution assumption is not met, even for logarithmic values. The extreme values identified have been discussed with experts (Eva Skiöldebrand and Elisabeth Hansson Rönnbäck). According to their assessment, these values are extreme but valid and they should thus be included in the analyzes. They also argue that these extreme values may be caused by a side-effect to the treatment in question, which is discussed in section 2.1.

1.4 Analysis software

R (version 4.0.0) has been used for all analyzes.

DS DS

2. PRIMARY RESULTS

In Tables 1 and 2 we see primary results for analyzes linked to the concentration of the biomarkers BGN 262 and COMP 1. The concentration is measured in the carpal joint (upper or middle joint compartment) that showed the highest concentration at visit 1 on verified lame leg. For both tables, the results of three tests are presented, one test per row in the table.

2.1 Concentration of BGN 262 in the carpal joint (upper or middle joint compartment) with the highest value at visit 1

Table 1 illustrates the results of the hypothesis tests regarding primary analyzes linked to the concentration of BGN 262 in synovial fluid. The first two hypothesis tests are to test if there is an effect on BGN 262 between visits 1 and 2 after treatment with drugs A and B, respectively. Here, the Wilcoxon signed rank test for paired samples has been used in both cases. The last row of the table refers to hypothesis tests if there is a difference in efficacy between the two treatment groups. In this case, the difference between visit 1 and visit 2 has been calculated for all horses and then the Wilcoxon rank sum test has been used to test whether the difference between visits 1 and 2 is systematically greater for either of the two groups.

Table 1. Results of hypothesis tests regarding the difference in concentration of BGN 262 in synovial fluid between visit 1 and visit 2, in the carpal joint (upper or middle joint compartment) with the highest concentration at visit 1

test	estimate	statistic	p.value	conf.low	conf.high	method	alternative	N
BGN 262, high, A	251,7	55	0,002(**)	142,656	342,275	Wilcoxon signed rank exact test	two.sided	10
BGN 262, high, B	-882,1	18	0,652	-2 977,988	318,397	Wilcoxon signed rank exact test	two.sided	9
BGN 262, high, A vs B	242,9	65	0,113	-44,394	2 634,926	Wilcoxon rank sum exact test	two.sided	(10, 9)

Test of Treatment group A

Since the p-value is lower than the significance level (0.05), we can reject the null hypothesis that the distribution for the concentration of BGN 262 is the same for visit 1 as for visit 2. There is a significant (p = 0.002(**)) decrease in concentration of BGN 262 between the two visits for those who were treated with treatment A.

Test of Treatment group B

For treatment group B, we see that the estimate is negative, which implies that the concentration on average has increased. However, we do not have enough support to reject the null hypothesis for this test. No significant difference can be demonstrated (P = 0.652).

Test for differences between treatment groups A and B

There is not enough statistical support to reject the null hypothesis, that the distributions for the difference in concentration between visits 1 and 2 are the same for the two treatment groups (A

DS
FB
DS
MP

DocuSign Envelope ID: 06A04576-8AB2-4997-AFCE-75ABF2223EF0

and B), $p = 0.113$. This result is, however, highly dependent on 3 extreme values in treatment group B and the mathematical properties associated with the rank sum test.

It is worth noting that all the extreme values are found in treatment group B.

From, among others, Sandstedt et al (2021) and Skiöldebrand et al (2017), it is concluded that biomarker COMP 1 is associated with a variety of damages and injuries, both in human and animal studies. It has therefore been suggested that the extreme values are the effect of cartilage destruction and a side-effect to the medication. Earlier studies of the BGN 262 and COMP 1 biomarkers, in which both healthy and injured horses have been compared, high concentrations have been associated to damages to joints. For example, Adepu et al (submitted, 2022), noted a significant and non-overlapping concentration of BGN 262 for horses with chip fracture joints, compared to healthy horses. Also, Ekman et al (2019) show that lame joints have COMP 1 concentrations generally 6 times higher compared to healthy, non-lame joints.

Having the assumption of side-effect in mind it could be assumed that the extreme values indicate pathologic degradation of cartilage and subchondral bone in the joint, and that they need to be separated from the other observations. The result from this study corroborates the possibility that there can be two groups of test subjects – comparable to what earlier studies have seen as a distinction between healthy and injured joints.

Although, it is not possible from these data alone to draw conclusions about the causal effects of treatment and inflicted damages, we find that the results can point in such a direction. Further studies, where the risk of damage is included in the primary analyzes plan and, if possible, covered by other methods of investigation is necessary to draw such conclusions. In such studies, cutoffs for BGN 262 and COMP 1 need to be specified in advance to make quantitative assessments on the prevalence of a possible risk for overreaction. Also, power and sample size estimations must take this possible reaction, and the necessary analyzes, into account.

DS DS

DocuSign Envelope ID: 06A04576-8AB2-4997-AFCE-75ABF2223EF0

2.2 Concentration of COMP 1 in the carpal joint (upper or middle joint compartment) with the highest value at visit 1

In Table 2 we see the results of the hypothesis tests regarding primary analyzes linked to the concentration of COMP 1 in synovial fluid. The first two hypothesis tests are to test for whether there is an effect on COMP 1 between visits 1 and 2 after treatment with drugs A and B respectively. Here, the Wilcoxon signed rank test for paired samples has been used in both cases. The last row of the table refers to hypothesis tests for whether there is a difference in efficacy between the two treatment groups. In this case, the difference between visit 1 and visit 2 has been calculated for all horses and then the Wilcoxon rank sum test has been used to test whether the difference between visits 1 and 2 is systematically greater for either of the two groups.

Table 2. Results of hypothesis tests regarding effect on concentration of COMP 1 in the carpal joint (upper or middle joint compartment) with the highest concentration at visit 1

test	estimate	statistic	p.value	conf.low	conf.high	method	alternative	N
Comp1, high, A	11,125	38,000	0,322	-9,454	29,436	Wilcoxon signed rank exact test	two.sided	10
Comp1, high, B	-62,007	0,000	0,002(**)	-2 514,110	-23,519	Wilcoxon signed rank exact test	two.sided	10
Comp 1, high, A vs B	58,798	94,000	0,000(***)	27,413	4 958,747	Wilcoxon rank sum exact test	two.sided	(10, 10)

Test of Treatment group A

For treatment group A, we do not have enough support to reject the null hypothesis that the distribution for concentration of COMP 1 is the same for visit 1 as for visit 2 ($p = 0.322$).

Test of Treatment group B

For the test that relates to treatment group B, we see that the estimate is negative, this means that the difference between visits 1 and 2 is negative and, in principle, that the concentration on average has increased. Since the p-value is lower than the significance level, we can reject the null hypothesis. There is a significant ($p = 0.002$(**)) increase in concentration of COMP 1 between the two visits for those who were treated with treatment B.

Test for difference in differences between treatment groups A and B

For the test regarding whether the difference in concentration of COMP 1 between visits 1 and 2 is the same for the two groups (A and B), we can reject the null hypothesis that the distributions for the difference between visits 1 and 2 are the same for the two groups. There is a significant difference between the groups regarding change in concentration between visits 1 and 2, ($p <$ **0.0005 (***))**.

DS FB DS MP

DocuSign Envelope ID: 06A04576-8AB2-4997-AFCE-75ABF2223EF0

3. SECONDARY RESULTS

In this Section, statistical analyzes are presented for the secondary outcomes.

3.1. Concentration of BGN 262 in synovial fluid in the middle joint compartment of the carpal joint

Tables 3 and 4 show the concentration of the biomarkers BGN 262 and COMP 1 in synovial fluid in the middle joint compartment of the carpal joint.

The first two hypothesis tests are to test for whether there is an effect on BGN 262 between visits 1 and 2 after treatment with drugs A and B, separately. Here, Wilcoxon signed rank test for paired samples has been used in both cases. The last row of the table refers to hypothesis tests for whether there is a difference in efficacy between the two treatment groups. In this case, the difference between visit 1 and visit 2 has been calculated for all horses and then the Wilcoxon rank sum test has been used to test whether the difference between visits 1 and 2 is systematically greater for either of the two groups.

Table 3. Results of hypothesis tests regarding effect on concentration of BGN 262 in synovial fluid in the middle joint compartment of the carpal joint.

test	estimate	statistic	p.value	conf.low	conf.high	method	alternative	N
BGN 262, mid, A	237,677	55,000	0,002(**)	142,656	343,526	Wilcoxon signed rank exact test	two.sided	10
BGN 262, mid, B	-704,928	16,000	0,496	-2 848,707	288,127	Wilcoxon signed rank exact test	two.sided	9
BGN 262, mid, A vs B	358,665	68,000	0,065	-21,930	2 260,846	Wilcoxon rank sum exact test	two.sided	(10, 9)

Treatment group A

Since the p-value is lower than the significance level, we can reject the null hypothesis that the distribution for concentration is the same for visit 1 as visit 2. There is a significant ($p = 0.002$(**)) decrease in concentration of BGN 262 between the two visits for those who were treated with treatment A.

Treatment group B

For the test that relates to treatment group B, we see that the estimate is negative, this means that the median difference between visits 1 and 2 is negative and that the concentration on average has increased. However, we do not have enough support to reject the null hypothesis for this test. No significant difference can be demonstrated, $p = 0.496$.

Test for differences between treatment groups A and B

DS FB DS MP

DocuSign Envelope ID: 06A04576-8AB2-4997-AFCE-75ABF2223EF0

We do not have enough support to reject the null hypothesis that the distribution for the difference in concentration between visits 1 and 2 is the same for the two groups (A and B), p = 0.065. The p-value is close to the significance limit 5%, which can be the effect of extreme values (see explanation for Table 1). Also, the sample size is rather small in this study, indicating a low power. This needs to be taken into consideration in the planning of future studies.

3.2 Concentration of COMP 1 in synovial fluid the middle joint compartment of the carpal joint

In Table 4 the results of the hypothesis tests regarding secondary analyzes linked to the concentration of COMP 1 in synovial fluid. The first two hypothesis tests are to test for whether there is an effect on COMP 1 between visits 1 and 2 after treatment with drugs A and B, separately. Here, the Wilcoxon signed rank test for paired samples has been used in both cases. The last row of the table refers to hypothesis tests for whether there is a difference in efficacy between the two treatment groups. In this case, the difference between visit 1 and visit 2 has been calculated for all horses and then the Wilcoxon rank sum test has been used to test whether the difference between visits 1 and 2 is systematically greater for either of the two groups.

Table 4. Results of hypothesis tests regarding effect on concentration of COMP 1 in synovial fluid the middle joint compartment of the carpal joint

test	estimate	statistic	p.value	conf.low	conf.high	method	alternative	N
Comp 1, mid, A	3,622	31,000	0,770	-13,242	19,345	Wilcoxon signed rank exact test	two.sided	10
Comp 1, mid, B	-417,114	2,000	0,006(**)	-2 509,466	-15,268	Wilcoxon signed rank exact test	two.sided	10
Comp 1, mid, A vs B	46,944	87,000	0,004(**)	14,891	1 464,031	Wilcoxon rank sum exact test	two.sided	(10,10)

Test of Treatment group A

For treatment group A and we do not have enough support to reject the null hypothesis that the distribution for concentration of COMP 1 is the same for visit 1 as for visit 2, p = 0.770.

Test of Treatment group B

For the test that relates to treatment group B, we see that the estimate is negative, this means that the median difference between visits 1 and 2 is negative and, in principle, that the concentration on average has increased. Since the p-value is lower than the significance level, we can reject the null hypothesis, the concentration at visit 2 is significantly higher than at visit 1 for group B, p =0.006 (**).

DS DS

DocuSign Envelope ID: 06A04576-8AB2-4997-AFCE-75ABF2223EF0

Test for differences between treatment groups A and B

For the test regarding whether the difference in concentration of COMP 1 between visits 1 and 2 is the same for the two groups (A and B), we can reject the null hypothesis that the distribution is the same. There is a significant difference between the groups regarding change in concentration between visits 1 and 2, p = 0.004 (**).

3.3 Comparison of the number of non-lame (lameness = 0 after flexion test) horses at visit 2

In Table 5, the number of horses per treatment group that are judged to be non-lame at visit 2. For treatment group B, only one horse is non-lame at visit 2.

Table 5. Table of the number of horses considered to be non-lame after flexion test at visit 2

Visit 2	Treatment A	Treatment B
Lame	5	9
Not lame	5	1

In Table 6 below, the results for hypothesis testing of the proportion of non-lame at visit 2 between treatment groups A and B. There is no support for rejecting the null hypothesis, there is no significant difference between the two groups, p = 0.141.

Table 6. Difference in proportion of non-lame horses between groups A and B after flexion test at visit 2

estimate	p.value	conf.low	conf.high	method	alternative
0,125	0,141	0,002	1,566	Fisher's Exact Test for Count Data	two.sided

DocuSign Envelope ID: 06A04576-8AB2-4997-AFCE-75ABF2223EF0

3.4 Comparison of the number of non-lame horses (lameness = 0 after flexion test) at visit 3

In Table 3 below, we see the number of horses considered to be non-lame after flexion tests at visit 3. Here we see that it is the same number for both groups.

Table 7. The number of horses that are non-lame or not after flexion test at visit 3

Non lame Visit 3	Treatment A	Treatment B
Lame	2	2
Not lame	8	8

Table 8 below shows the result of the hypothesis test and as expected, there is no support to reject the null hypothesis that the proportions for A and B are the same, p =1.

Table 8. Difference in proportion of non-lame horses between groups A and B after flexion test at visit 3

estimate	p.value	conf.low	conf.high	method	alternative
1.000	1.000	0.059	17.078	Fisher's Exact Test for Count Data	two.sided

3.5 Comparison of the number of non-lame horses (after flexion test) at both visits 2 and 3

Table 9 below shows the possible combinations for whether a horse is non-lame or not at visits 2 and 3 for treatment group A. For example, it shows that half of the horses in the treatment group are non-lame at both visits and that there is no horse that has gone from being non-lame at visit two to being assessed as lame at visit 3.

Table 9. Number of horses per possible combination regarding non-lame or not after flexion test for visits 2 and 3, treatment group A

Visit 2	Visit 3	Count
Lame	Lame	2
Not lame	Lame	0
Lame	Not lame	3
Not lame	Not lame	5

DS FB DS MP

DocuSign Envelope ID: 06A04576-8AB2-4997-AFCE-75ABF2223EF0

Table 10 below shows the possible combinations for whether a horse is lame or not at visits 2 and 3 for treatment group B. Here, most horses are lame at visit 2 but non-lame at visit 3.

Table 10. Number of horses per possible combination regarding non-lame or not after flexion test for visits 2 and 3, treatment group B

Visit 2	Visit 3	Count
Lame	Lame	1
Not lame	Lame	1
Lame	Not lame	8
Not lame	Not lame	0

Table 11. Table for the number of horses assessed as non-lame at both visits for the two treatment groups

Not lame both visits	Treatment A	Treatment B
No	5	10
Yes	5	0

Table 12 below presents the results for hypothesis tests regarding the proportion of horses assessed as non-lame at both visits (2 and 3) between the treatment groups. Since the p-value is below the significance level, we can reject the null hypothesis and the difference in the proportion between the two groups is significant.

Table 12. Hypothesis test for difference between groups A and B regarding the proportion of horses assessed as non-lame at both visits 2 and 3

estimate	p.value	conf.low	conf.high	method	alternative
0.000	0.033(*)	0.000	0.837	Fisher's Exact Test for Count Data	two.sided

DocuSign Envelope ID: 06A04576-8AB2-4997-AFCE-75ABF2223EF0

3.6 Results for blood samples analysis

Results of blood test analyzes are reported separately.

REFERENCES

- Adepu S, Ekman S, Leth J, Johansson U. Lindahl A. Skiöldebrand S. (2022). *Biglycan neo-epitope (BGN262), a Novel biomarker for screening early changes in equine osteoarthritic subchondral bone*. Under revision in Osteoarthritis and Cartilage.
- Ekman, S., Lindahl, A., Rüetschi, U., Jansson, A., Björkman, K., Abrahamsson-Aurell, K., Björnsdóttir, S., Löfgren, M., Hultén, L.M. and Skiöldebrand, E. (2019). *Effect of circadian rhythm, age, training and acute lameness on serum concentrations of cartilage oligomeric matrix protein (COMP) neo-epitope in horses*. Equine Vet J, 51: 674-680. https://doi.org/10.1111/evj.13082
- Sandstedt, J., K. Vargmar, K. Björkman, U. Ruetschi, G. Bergström, L. Mattsson Hultén, E. Skiöldebrand (2021). *COMP (Cartilage Oligomeric Matrix Protein) – Neoepitope A Novel Biomarker to Identify Symptomatic Carotid Stenosis*, Arterioscler Thromb Vasc Biol. 2021;41:1218–1228. DOI: 10.1161/ATVBAHA.120.314720
- Skiöldebrand, E., S. Ekman, L. Mattsson Hultén, E. Svala, K. Björkman, A. Lindahl, A. Lundqvist, P. Önnerfjord, C. Sihlbom and U. Rüetschi (2017). *Cartilage oligomeric matrix protein neoepitope in the synovial fluid of horses with acute lameness: A new biomarker for the early stages of osteoarthritis*, Equine Veterinary Journal 49, pp 662–667

DS FB DS MP

DocuSign Envelope ID: 06A04576-8AB2-4997-AFCE-75ABF2223EF0

APPENDIX A. NORMAL DISTRIBUTION TEST

Tests for normal distribution of residuals for linear mixed models are shown in Table A 1 below. The null hypothesis is that the data come from a normally distributed population. Below we reject the null hypothesis that the residuals are normally distributed in all cases.

Table A 1. Normal distribution test of residuals for linear mixed models for all variables related to biomarker concentration

Test	statistic	p.value	method
residuals marginal BGN high log	0.828	0.000(***)	Shapiro-Wilk normality test
residuals ss BGN high log	0.932	0.021(*)	Shapiro-Wilk normality test
residuals marginal BGN mid log	0.907	0.003(**)	Shapiro-Wilk normality test
residuals ss BGN mid log	0.907	0.003(**)	Shapiro-Wilk normality test
residuals marginal COMP high log	0.736	0.000(***)	Shapiro-Wilk normality test
residuals ss COMP high log	0.934	0.021(*)	Shapiro-Wilk normality test
residuals marginal COMP mid log	0.813	0.000(***)	Shapiro-Wilk normality test
residuals ss COMP mid log	0.813	0.000(***)	Shapiro-Wilk normality test

Accounting for p-values for testing the difference of the logarithmic concentrations between visits 1 and 2 for the two biomarkers is shown in the Table below. Here it can be noted that the null hypothesis cannot be rejected for BGN 262. However, the assessment has been made to still apply a nonparametric test without assuming distribution as the p-values are relatively low and the result above indicates non-normal distributed residuals. Also, for the sake of comparability, the same methods have been applied to all concentrations of biomarkers in synovial fluid.

DS DS

DocuSign Envelope ID: 06A04576-8AB2-4997-AFCE-75ABF2223EF0

Table A 2. Normal distribution test for differences in concentration between visits 1 and 2, the tests are done on logarithmic values

Test	statistic	p.value	method
BGN 262 high log	0.904	0.058	Shapiro-Wilk normality test
BGN 262 mid log	0.915	0.090	Shapiro-Wilk normality test
COMP1 high log	0.766	0.000(***)	Shapiro-Wilk normality test
COMP1 mid log	0.824	0.002(**)	Shapiro-Wilk normality test

DS DS

DocuSign Envelope ID: 06A04576-8AB2-4997-AFCE-75ABF2223EF0

APPENDIX B. DEMOGRAPHIC DATA AND BASELINE VALUES

Table B 1. Demographic data by group and at the overall level

	Treatment A (N = 10)	*Treatment B (N = 10)*	*Total (N = 20)*
Age			
min:	2	2	2
mean (sd)	3.50 (1.84)	4.50 (2.37)	4.00 (2.13)
median (IQR)	3.00 (2.25, 3.75)	5.00 (2.00, 6.00)	3.00 (2.00, 6.00)
Max:	8	8	8
Sex			
Mare, n (%)	2 (20)	1 (10)	3 (15)
Stallion, n (%)	4 (40)	4 (40)	8 (40)
Gelding, n (%)	4 (40)	5 (50)	9 (45)

Table B 2. Baseline values for variables linked to lameness

	Treatment A (N = 10)	*Treatment B (N = 10)*	*Total (N = 20)*
Qscore visit 1			
min:	0	0	0
mean (sd)	13.61 (5.74)	10.67 (9.27)	12.14 (7.65)
median (IQR)	14.35 (12.30, 16.02)	12.40 (1.18, 16.38)	13.55 (9.05, 16.33)
Max:	21.4	27.5	27.5
>0, n (%)	9 (90)	7 (70)	16 (80)
Flexion test visit 1			
min:	1	1	1
mean (sd)	1.50 (0.41)	1.40 (0.39)	1.45 (0.39)
median (IQR)	1.50 (1.12, 1.88)	1.50 (1.00, 1.50)	1.50 (1.00, 1.62)
Max:	2	2	2
>0, n (%)	10 (100)	10 (100)	20 (100)

DS FB DS MP

DocuSign Envelope ID: 06A04576-8AB2-4997-AFCE-75ABF2223EF0

Table B 3. Starting values for concentration of biomarkers per group. The concentration of BGN 262 and COMP 1 is quantified in synovial fluid from the carpal joint (upper or middle joint compartment). The highest value is obtained from either the upper or the middle joint compartment.

	Group A (N = 10)	*Group B (N = 10)*	*Total (N = 20)*
BGN 262 (highest value) visit 1			
min:	351.38	304.52	304.52
mean (sd)	543.93 (140.89)	524.33 (140.26)	534.13 (137.19)
median (IQR)	539.89 (425.70, 625.50)	548.44 (430.96, 567.52)	548.44 (407.61, 610.33)
Max:	754.2	774.5	774.5
BGN 262 (middle joint) visit 1			
min:	351.38	118.45	118.45
mean (sd)	518.81 (132.99)	465.85 (194.62)	492.33 (164.49)
median (IQR)	495.88 (414.55, 614.74)	512.43 (317.54, 555.65)	509.06 (389.28, 578.05)
Max:	754.2	774.5	774.5
COMP 1 (highest value) visit 1			
min:	18.85	13.96	13.96
mean (sd)	41.84 (16.17)	30.00 (11.47)	35.92 (14.94)
median (IQR)	41.70 (27.82, 57.42)	26.76 (22.39, 36.41)	36.14 (23.56, 47.34)
Max:	61.29	52.95	61.29
COMP 1 (middle joint) visit 1			
min:	14.93	12.17	12.17
mean (sd)	26.05 (12.32)	25.78 (13.85)	25.92 (12.76)
median (IQR)	19.68 (17.95, 35.68)	23.41 (18.30, 26.88)	21.45 (17.93, 28.17)
Max:	46.66	61.29	61.29

DS DS

DocuSign Envelope ID: 06A04576-8AB2-4997-AFCE-75ABF2223EF0

APPENDIX C. FIGURES FOR CONCENTRATION OF BIOMARKERS

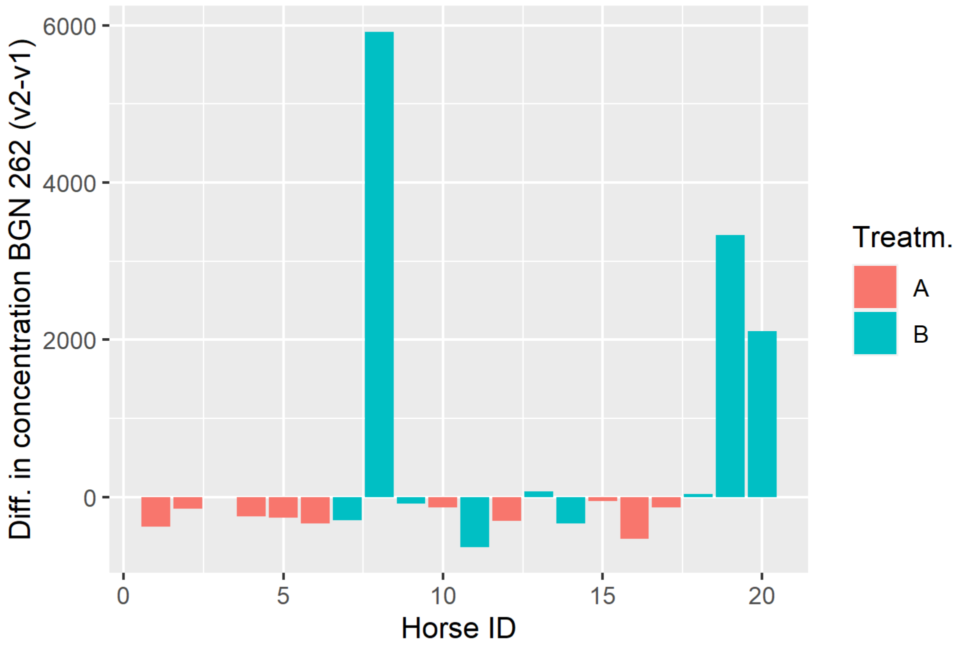

Figure C 1. Differences in concentration of BGN 262 in synovial fluid in the carpal joint (upper or middle joint compartment) that had the highest concentration at visit 1 (concentration for visit 2 - concentration for visit 1).

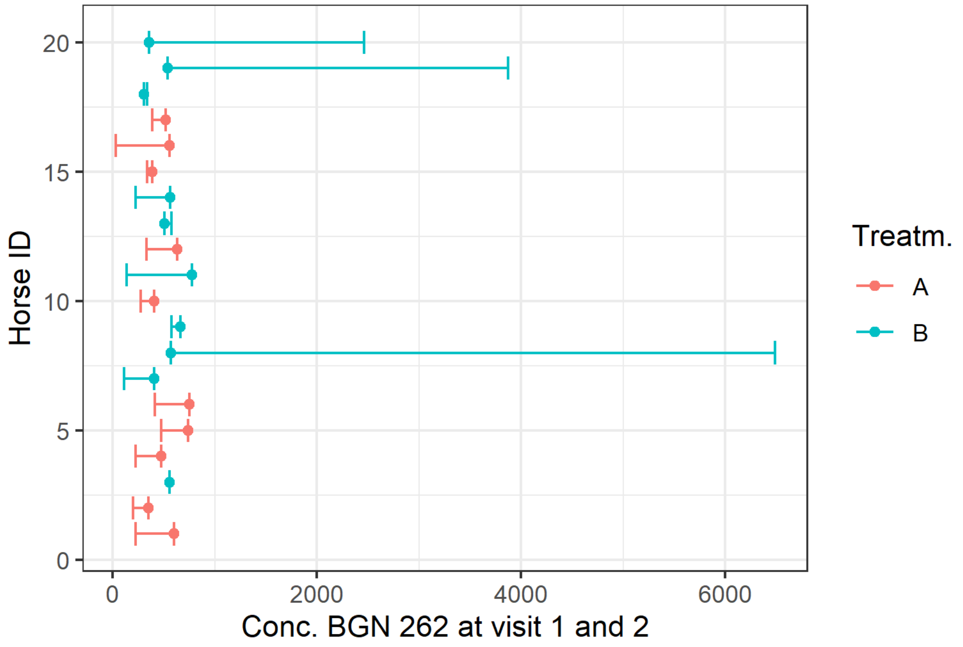

Figure C 2. The concentration of BGN 262 in the synovial fluid in the carpal joint (upper or middle joint compartment) that had the highest concentration at visit 1. The round marker shows the value of visit 1 and the vertical line shows the value of visit 2.

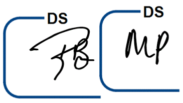

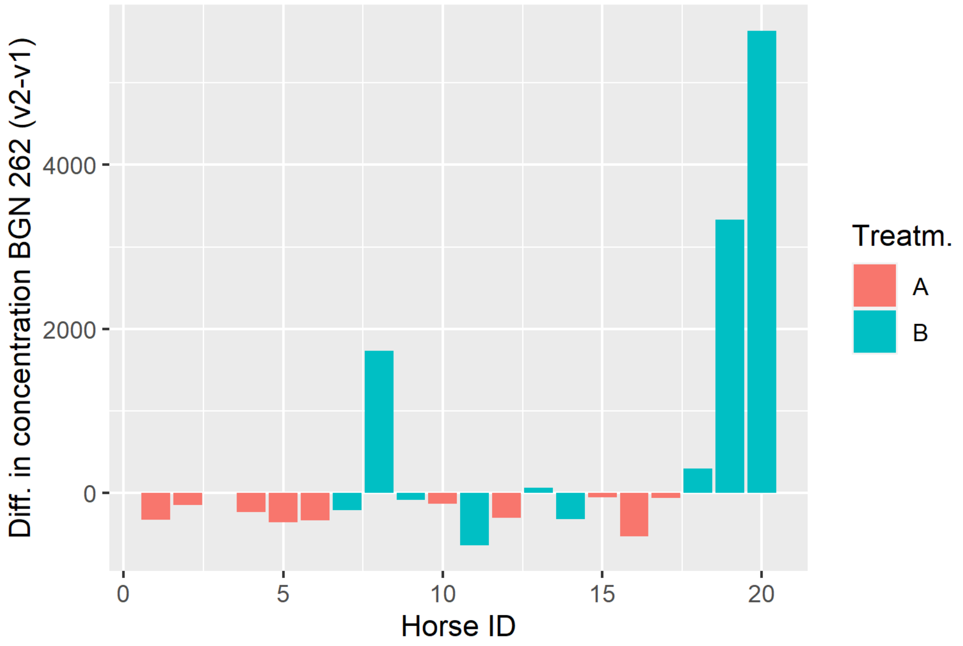

Figure C 3. Differences in concentration of BGN 262 in synovial fluid in the middle joint compartment of the carpal joint (concentration for visit 2 - concentration for visit 1).

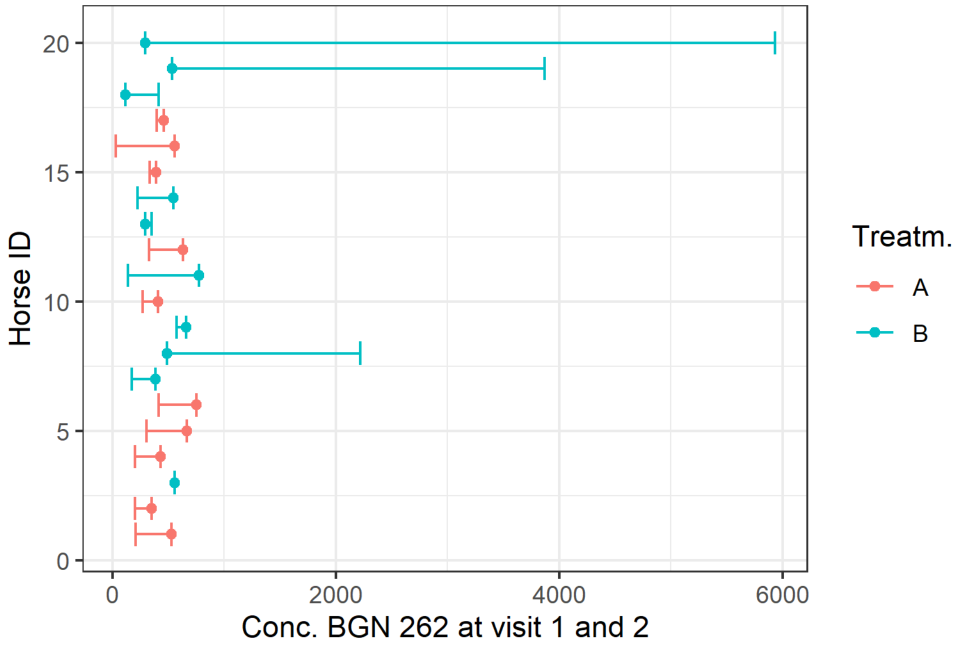

Figure C 4. The concentration of BGN 262 in the synovial fluid in the middle joint compartment of the carpal joint at visit 1. The round marker shows the value for visit 1 and the vertical line shows the value at visit 2.

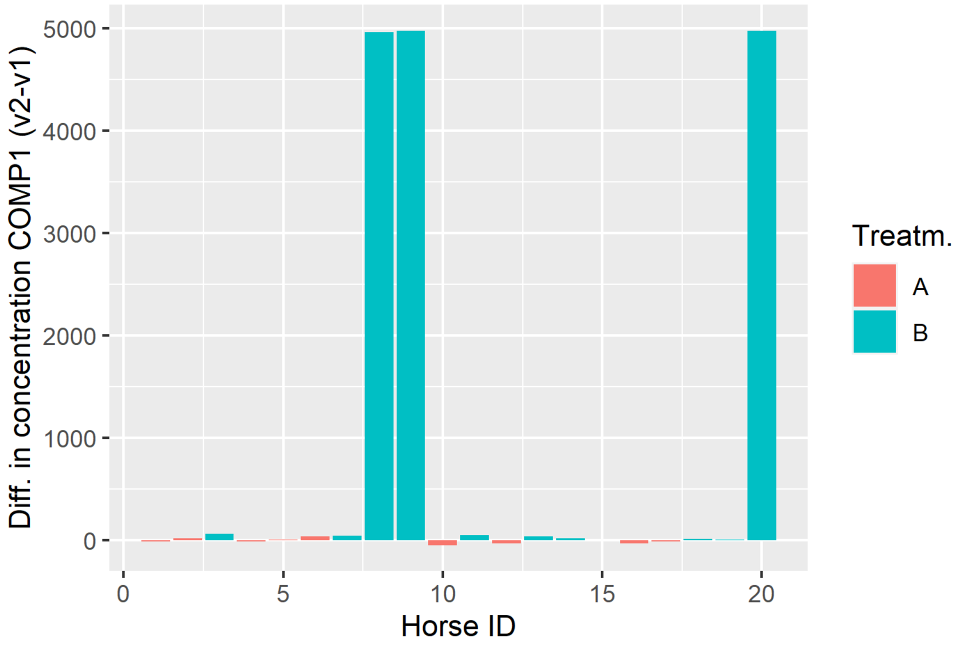

Figure C 5. Differences in concentration of COMP 1 in synovial fluid in the carpal joint (upper or middle joint compartment) that had the highest concentration at visit 1 (concentration for visit 2 - concentration for visit 1).

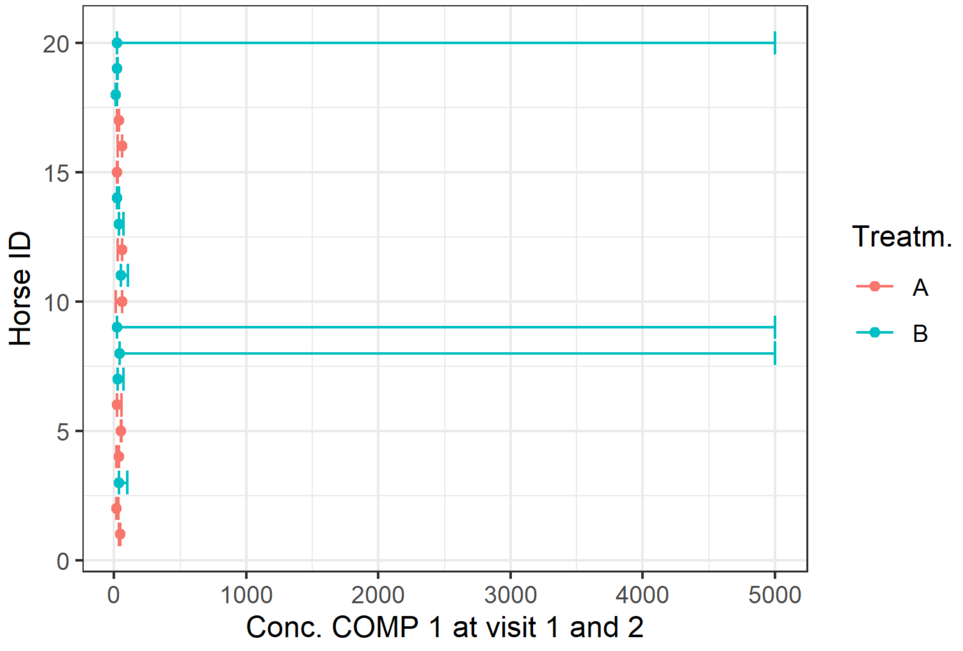

Figure C 6. The concentration of COMP 1 in the synovial fluid in the carpal joint (upper or middle joint compartment) that had the highest concentration at visit 1. The round marker shows the value of visit 1 and the vertical line shows the value of visit 2.

DocuSign Envelope ID: 06A04576-8AB2-4997-AFCE-75ABF2223EF0

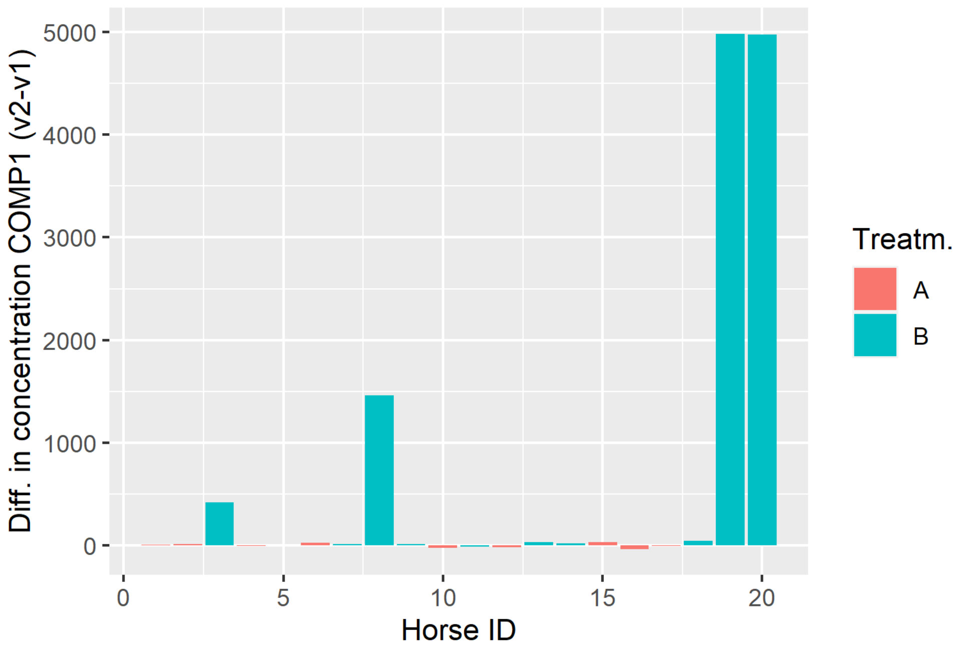

Figure C 7. Differences in concentration of COMP 1 in the middle joint compartment of the carpal joint (concentration for visit 2 - concentration for visit 1).

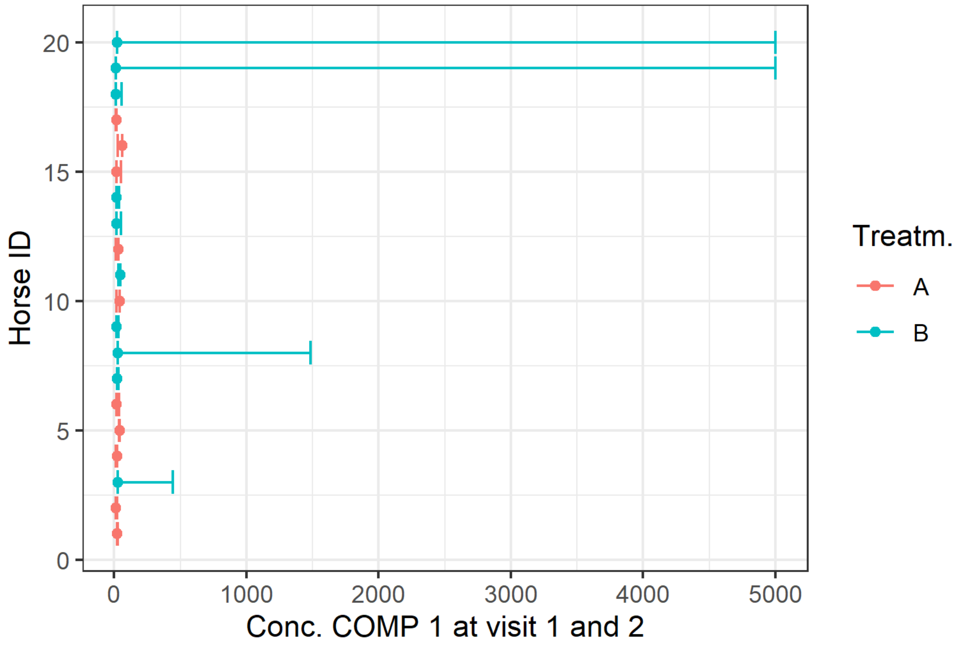

Figure C 8. The concentration of COMP 1 in the middle joint compartment of the carpal joint. The round marker shows the value of visit 1 and the vertical line shows the value of visit 2.

DS DS

DocuSign Envelope ID: 06A04576-8AB2-4997-AFCE-75ABF2223EF0

Statistikkonsulterna
JOSTAT & MR SAMPLE AB

Statistical analyzes and results of explorative outcomes

Study title: Intra articular treatment with a new drug combination in comparison with Celeston Bifas in horses with lameness associated with osteoarthritis (OA-252)

**Date:**	**Authors:**
2022-06-22	Magnus Pettersson
	DocuSigned by: Magnus Pettersson 2B47B83A416F4A2...
**Status:**	Rebecca Bylund
Final	DocuSigned by: Rebecca Bylund 69BAF243696D455...

Summary

This report contains results for explorative outcomes in accordance with SAP for the study "*Intra-articular treatment with a new drug combination in comparison with Celeston bifas in horses with lameness associated with Osteoarthritis*". This is the second of two reports, where part 1 contains results for the primary and secondary outcomes.

DocuSign Envelope ID: 06A04576-8AB2-4997-AFCE-75ABF2223EF0

TABLE OF CONTENTS

Summary....1

1. Assumptions and statistical methods 3

1.1 Assessment of distributions.... 3

1.2 Handling of extreme values.... 3

1.3 Analysis software 3

2. Results 3

2.1 Lameness locator (Q-score).... 3

2.2 Concentration of BGN 262 in the carpal joint (upper or middle joint compartment) that showed the lowest concentration at visit 1 5

2.3 Concentration of COMP 1 in the carpal joint (upper or middle joint compartment) that showed the lowest concentration at visit 1 6

2.4 Lameness score after flexion test.... 7

2.5 Concentration of BGN 262 in serum.... 10

2.6 Concentration of COMP 1 in serum.... 13

Appendix a. Tests of normality assumptions 16

Appendix b. Baseline values and demographics 17

Appendix c. Figures for concentration of biomarkers 18

DS DS

DocuSign Envelope ID: 06A04576-8AB2-4997-AFCE-75ABF2223EF0

1. ASSUMPTIONS AND STATISTICAL METHODS

1.1 Assessment of distributions

Assessment of the normal distribution assumption for the concentration of biomarkers (BGN 262 and COMP 1) in serum has been made for both non-logarithmic and logarithmic values. The normal distribution assumption has been evaluated by analyzing the distribution of the error terms for linear mixed models. Data seemed normally distributed when visualized, so models were fitted, and the distribution of the residuals were assessed. Shapiro Wilk's test has been applied and the results are presented in Appendix A. In short, the analyzes show that data can be assumed to be normally distributed.

The distribution for Q-score and clinical lameness were assessed for each visit. The data could not be assumed to be normally distributed. The treatment effect on clinical lameness after flexion test and Q-score were assessed visually and through descriptive statistics.

For the concentration of the biomarkers in synovial fluid Wilcoxon signed rank test and Wilcoxon rank sum test was used due to non-normality of the data and to enhance comparability with primary and secondary results. For these methods, the exact same results are obtained for logarithmic and non-logarithmic values as the tests are rank based. Estimates and confidence intervals for original (non-logarithmic) values are also presented.

1.2 Handling of extreme values

For the concentration of the biomarkers BGN 262 and COMP 1 in synovial fluid in the carpal joint(upper or middle joint compartment) that showed the lowest concentration at visit 1, there are values that appear extreme in relation to the others for visit 2. There are two horses where the concentration of the biomarker in question has increased dramatically between visits 1 and 2. It is the same two horses for both biomarkers. Appendix C includes figures showing the difference in concentration between visit 2 and visit 1 where the two outliers are apparent.

These values may be a reason why the normal distribution assumption is not met, even for logarithmic values. The extreme values identified have been discussed with experts (Eva Skiöldebrand and Elisabeth Hansson Rönnbäck). According to their assessment, these values are extreme but valid and they should thus be included in the analysis. They also argue that these extreme values may be caused by an adverse event to the treatment in question. This is discussed further in Statistical report 1.

1.3 Analysis software

R (version 4.0.0) has been used for all analyzes.

2. RESULTS

2.1 Lameness locator (Q-score)

Comparing Q-score for the three visits between group A and group B in table 1, shows that although the median Q-score for visit 1 is lower for group B there is more variety in group B than in A. The inter quartile range (IQR) for group B is (1.18, 16.38) while it is (12.30, 16.02) for group A. The median value decreases steadily for group A but the total range as well as the maximum

DS FB DS MP

DocuSign Envelope ID: 06A04576-8AB2-4997-AFCE-75ABF2223EF0

level increases. For group B the median drops from 12.40 to 2 between visit 1 and 2 but then increases to 5.35 at visit 3.

Table 1. Descriptive statistics of the Q-score for each visit and treatment group as well as for the whole sample

	A (N = 10)	B (N = 10)	Total (N = 20)
**Q-score visit 1**			
**min:**	0	0	0
**mean (sd)**	13.61 (5.74)	10.67 (9.27)	12.14 (7.65)
**median (IQR)**	14.35 (12.30, 16.02)	12.40 (1.18, 16.38)	13.55 (9.05, 16.33)
**max:**	21.4	27.5	27.5
**>0, n (%)**	9 (90)	7 (70)	16 (80)
**Q-score visit 2**			
**min:**	0	0	0
**mean (sd)**	9.03 (6.96)	8.92 (12.01)	8.97 (9.56)
**median (IQR)**	10.15 (2.05, 13.47)	2.00 (0.00, 16.95)	8.45 (0.00, 14.62)
**max:**	17.9	29.4	29.4
**>0, n (%)**	7 (70)	5 (50)	12 (60)
**Q-score visit 3**			
**min:**	0	0	0
**mean (sd)**	8.05 (7.72)	5.08 (5.09)	6.57 (6.54)
**median (IQR)**	8.75 (0.00, 13.92)	5.35 (0.00, 8.30)	5.75 (0.00, 11.75)
**max:**	19.9	13.4	19.9
**>0, n (%)**	6 (60)	6 (60)	12 (60)

These results are also displayed in figure 1.

DS DS

DocuSign Envelope ID: 06A04576-8AB2-4997-AFCE-75ABF2223EF0

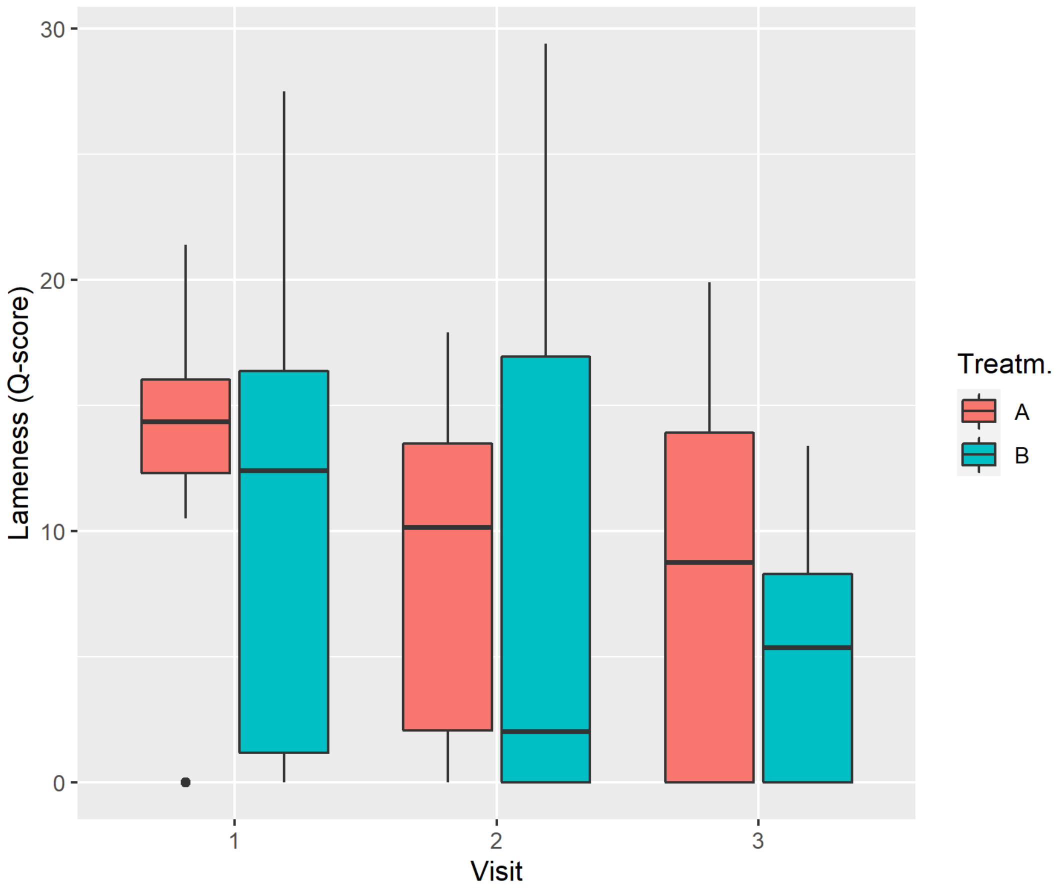

Figure 1. Boxplots for the distribution of Q-score at each visit for treatment A and B

2.2 Concentration of BGN 262 in the carpal joint (upper or middle joint compartment) that showed the lowest concentration at visit 1

In Table 2, the results of the hypothesis tests regarding explorative analyzes linked to the concentration of BGN 262 in synovial fluid are shown. The first two hypothesis tests are to test for whether there is an effect on BGN 262 between visits 1 and 2 after treatment with drugs A and B respectively. Here, the Wilcoxon signed rank test for paired samples has been used in both cases. The last row of the table refers to hypothesis tests for whether there is a difference in efficacy between the two treatment groups. In this case, the difference between visit 1 and visit 2 has been calculated for all horses and then the Wilcoxon rank sum test has been used to test whether the difference between visits 1 and 2 is systematically greater for either of the two groups.

DS
DS

DocuSign Envelope ID: 06A04576-8AB2-4997-AFCE-75ABF2223EF0

Table 2. Test results for Wilcoxon tests for differences in concentration of BGN 262 in synovial fluid between visit 1 and 2 in the carpal joint (upper or middle joint compartment) that showed the lowest concentration at visit 1

test	estimate	statistic	p.value	conf.low	conf.high	method	alternative
BGN 262, Low, A	95,403	42,000	0,160	-44,488	232,369	Wilcoxon signed rank exact test	two.sided
BGN 262, Low, B	-287,028	0,000	0,002	-2 848,707	-142,061	Wilcoxon signed rank exact test	two.sided
BGN 262, Low, A vs B	388,131	91,000	0,001	168,794	690,356	Wilcoxon rank sum exact test	two.sided

Test of Treatment group A

There is not enough evidence to reject the null hypothesis that the distribution for the concentration of BGN 262 is the same for visit 1 as for visit 2 (p = 0.160).

Test of Treatment group B

For treatment group B, the estimate is negative, which implies that the concentration on average has increased. In this case we have a significant result (p = 0.002) which indicates that there is a significant increase in BGN 262 concentration in synovial fluid between visit 1 and 2 for group B.

Test for difference between treatment groups A and B

For the test of differences between the two groups we can reject the null hypothesis (p = 0.001) that the distributions for the difference in concentration between visits 1 and 2 are the same for the two treatment groups (A and B) which indicates that there is a significant difference between the groups.

2.3 Concentration of COMP 1 in the carpal joint (upper or middle joint compartment) that showed the lowest concentration at visit 1

In Table 3 the results of the hypothesis tests regarding explorative analyzes linked to the concentration of COMP 1 in synovial fluid are presented. The first two hypothesis tests are to test for whether there is an effect on COMP 1 between visits 1 and 2 after treatment with drugs A and B respectively. In these cases, the Wilcoxon signed rank test for paired samples has been used. The last row of the table refers to hypothesis tests for whether there is a difference in efficacy between the two treatment groups. In this case, the difference between visit 1 and visit 2 has been calculated for all horses and then the Wilcoxon rank sum test has been used to test whether the difference between visits 1 and 2 is systematically greater for either of the two groups.

DocuSign Envelope ID: 06A04576-8AB2-4997-AFCE-75ABF2223EF0

Table 3. Test results for Wilcoxon tests for differences in concentration of COMP 1 in synovial fluid between visit 1 and 2 in the carpal joint (upper or middle joint compartment) that showed the lowest concentration at visit 1

test	estimate	statistic	p.value	conf.low	conf.high	method	alternative
Comp 1, Low A	-1,404	23,000	0,695	-14,500	6,725	Wilcoxon signed rank exact test	two.sided
Comp 1, Low, B	-212,953	0,000	0,002	-2 514,285	-27,513	Wilcoxon signed rank exact test	two.sided
Comp 1, Low, A vs B	47,356	94,000	0,000	23,190	420,711	Wilcoxon rank sum exact test	two.sided

Test of Treatment group A

For treatment group A, we do not have enough support to reject the null hypothesis that the distribution for concentration of COMP 1 is the same for visit 1 as for visit 2 ($p = 0.695$).

Test of Treatment group B

For the test that relates to treatment group B, the estimate is negative, this means that the difference between visits 1 and 2 is negative and, in principle, that the concentration on average has increased. Since the p-value is lower than the significance level, we can reject the null hypothesis. There is a significant ($p = 0.002$(**)) increase in concentration of COMP1 in synovial fluid between the two visits for those who were treated with treatment B.

Test for difference between treatment groups A and B

For the test regarding whether the difference in concentration of COMP 1 between visits 1 and 2 is the same for the two groups (A and B), we can reject the null hypothesis that the distributions for the difference between visits 1 and 2 are the same for the two groups. There is a significant difference between the groups regarding change in concentration between visits 1 and 2, $p < 0.0005$ (***).

2.4 Lameness score after flexion test

In table 4 below summary statistics for the clinical lameness score after flexion test, between the visits are displayed. The two groups start off with the same median (1.5) and by visit 2 it has dropped to 0.25 for group A and to 1 for group B. By visit 3 it has dropped to 0 for both groups.

DS FB
DS MP

DocuSign Envelope ID: 06A04576-8AB2-4997-AFCE-75ABF2223EF0

Table 4. Summary statistics for the clinical lameness score for the three visits displayed by treatment group and total sample

	A (N = 10)	B (N = 10)	Total (N = 20)
**Clinical lameness visit 1**			
**min:**	1	1	1
**mean (sd)**	1.50 (0.41)	1.40 (0.39)	1.45 (0.39)
**median (IQR)**	1.50 (1.12, 1.88)	1.50 (1.00, 1.50)	1.50 (1.00, 1.62)
**max:**	2	2	2
**>0, n (%)**	10 (100)	10 (100)	20 (100)
**Clinical lameness visit 2**			
**min:**	0	0	0
**mean (sd)**	0.50 (0.58)	0.80 (0.35)	0.65 (0.49)
**median (IQR)**	0.25 (0.00, 1.00)	1.00 (0.62, 1.00)	1.00 (0.00, 1.00)
**max:**	1.5	1	1.5
**>0, n (%)**	5 (50)	9 (90)	14 (70)
**Clinical lameness visit 3**			
**min:**	0	0	0
**mean (sd)**	0.15 (0.34)	0.10 (0.21)	0.12 (0.28)
**median (IQR)**	0.00 (0.00, 0.00)	0.00 (0.00, 0.00)	0.00 (0.00, 0.00)
**max:**	1	0.5	1
**>0, n (%)**	2 (20)	2 (20)	4 (20)

In figure 2 below these results are visualized in boxplots. It appears that most horses in group A decrease their score faster than in group B. However, there is a wider range of values in group A for visit 2.

DS FB
DS MP

DocuSign Envelope ID: 06A04576-8AB2-4997-AFCE-75ABF2223EF0

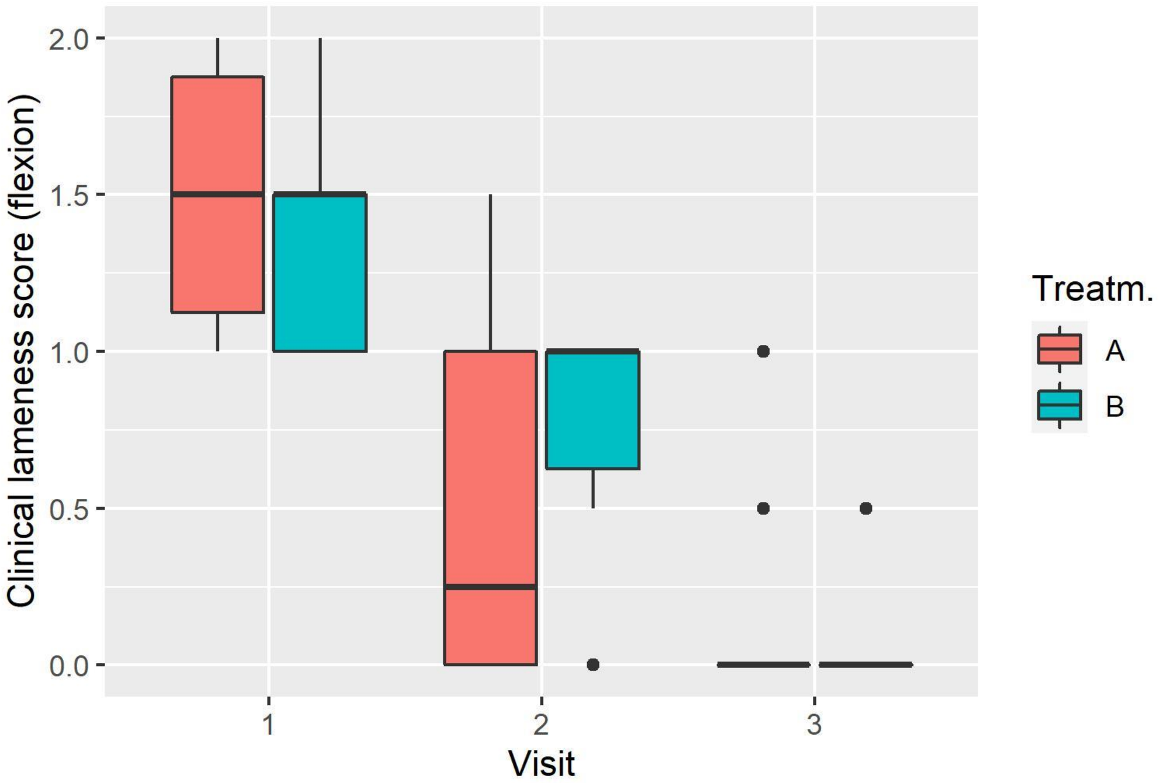

Figure 2. Boxplots for distribution of clinical lameness score after flexion test at each visit for treatment A and B

To clarify this further, Sankey plots of the movements between different lameness scores were made. These plots illustrate the number of horses that move from one score to another between each visit for each of the two treatment groups, one at a time.

DocuSign Envelope ID: 06A04576-8AB2-4997-AFCE-75ABF2223EF0

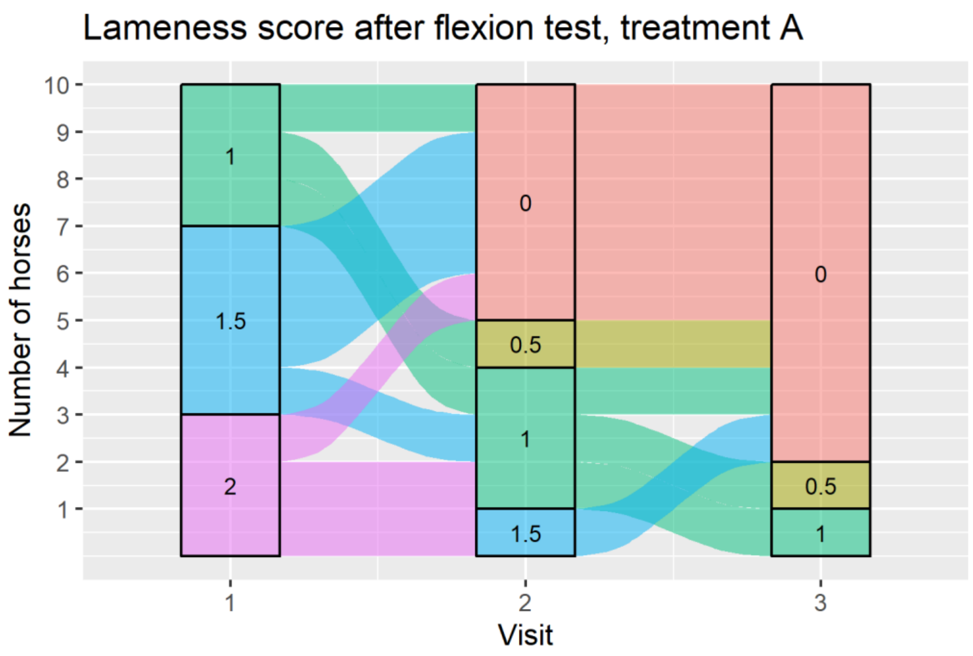

Figure 3. Sankey diagram illustrating clinical lameness scores after flexion test for visit 1, 2 and 3 for treatment group A

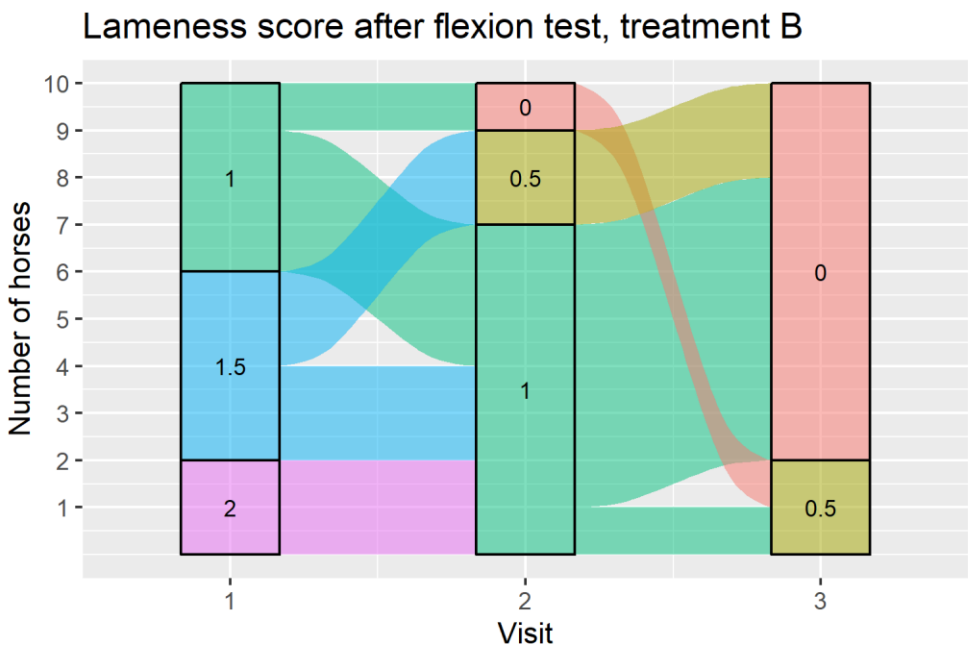

Figure 4. Sankey diagram illustrating clinical lameness scores after flexion test for visit 1, 2 and 3 for treatment group B

2.5 Concentration of BGN 262 in serum

To assess the effect on the concentration of BGN 262 in serum between visit 1, 2 and 3 and potential differences between the groups a linear mixed model was fitted. In the model, visit and

DS DS

DocuSign Envelope ID: 06A04576-8AB2-4997-AFCE-75ABF2223EF0

treatment group were modelled as fixed effects and the intercept was modelled as a random effect. The correlation between the observations was modelled as an autoregressive process, assuming that the error terms for an individual are related to its own past values and that the correlation declines exponentially with the number of periods separating them. The model was fitted on log transformed values. The estimates for the fixed effects are presented in table 5. Note that the effect (coefficient) for treatment group is specified for treatment B in relation to treatment A, hence treatment A is not present in the table.

Table 5. Model summary for fixed effects in linear mixed model for BGN 262 concentration in serum

	COEF	SE	Wald	P-value
**Visit**	-0.0273	0.0183	-1.492	0.136
**Treatment B**	-0.0982	0.0716	-1.372	0.170

There are no significant effects for either visit (slope) or treatment group. The individual trajectories for the concentrations are displayed in figure 5, here it is also evident that there are no clear group-specific patterns or overall patterns that stand out.

DS FB DS MP

DocuSign Envelope ID: 06A04576-8AB2-4997-AFCE-75ABF2223EF0

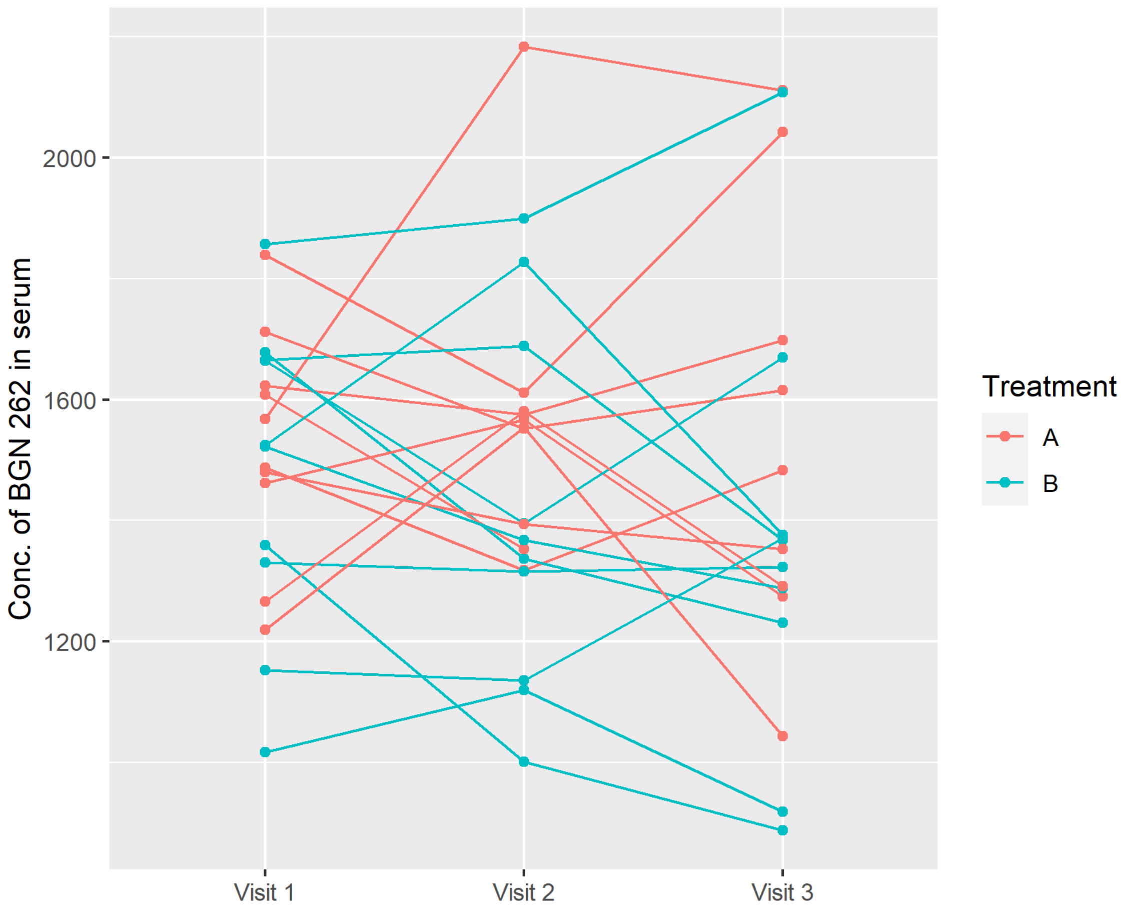

Figure 5. Line chart illustrating individual trajectories for concentration of BGN 262 in serum at visit 1, 2 and 3

DS
DS

DocuSign Envelope ID: 06A04576-8AB2-4997-AFCE-75ABF2223EF0

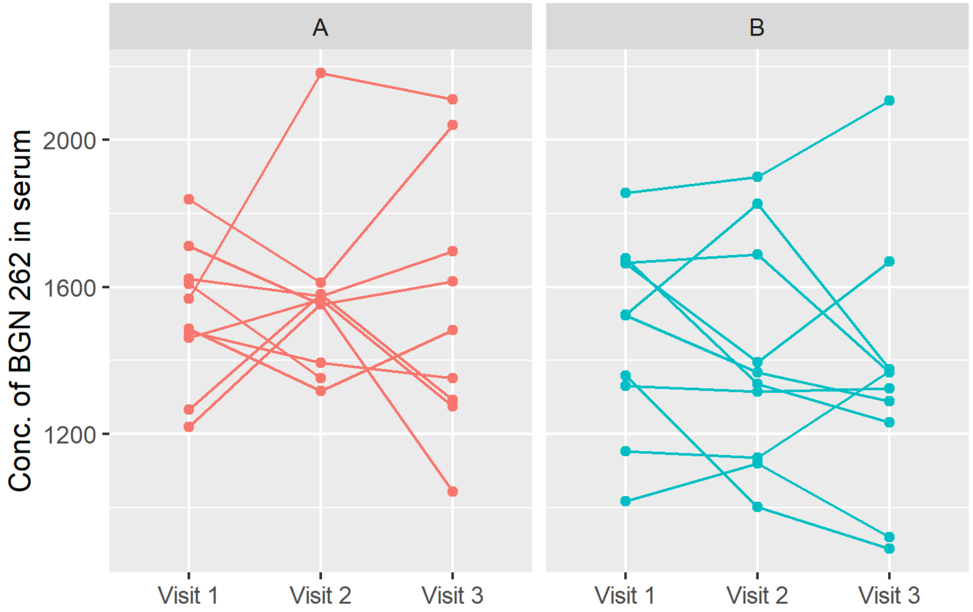

Figure 6. Line chart illustrating individual trajectories for concentration of BGN 262 in serum for each treatment group separately

2.6 Concentration of COMP 1 in serum

To assess the concentration of COMP 1 in serum between visit 1, 2 and 3 and potential differences between the groups a linear mixed model was fitted. In the model, visit and treatment group were modelled as fixed effects and the intercept was modelled as a random effect. The correlation between the observations was modelled as an autoregressive process, assuming the error terms for an individual are related to its own past values and that the correlation declines exponentially with the number of periods separating them. The model was fitted on log transformed values. The estimates for the fixed effects are presented in table 6. Note that the effect (coefficient) for treatment group is specified for treatment B in relation to treatment A, hence treatment A is not present in the table.

Table 6. Model summary for fixed effects in linear mixed model for COMP 1 concentration in serum

	COEF	SE	Wald	P-value
**Visit**	0.067	0.043	1.540	0.123
**Treatment B**	0.031	0.127	0.244	0.808

The individual trajectories for the concentrations are displayed in figure 6, again it is evident that there are no clear group-specific patterns or overall patterns of change that stand out.

DS
DS

DocuSign Envelope ID: 06A04576-8AB2-4997-AFCE-75ABF2223EF0

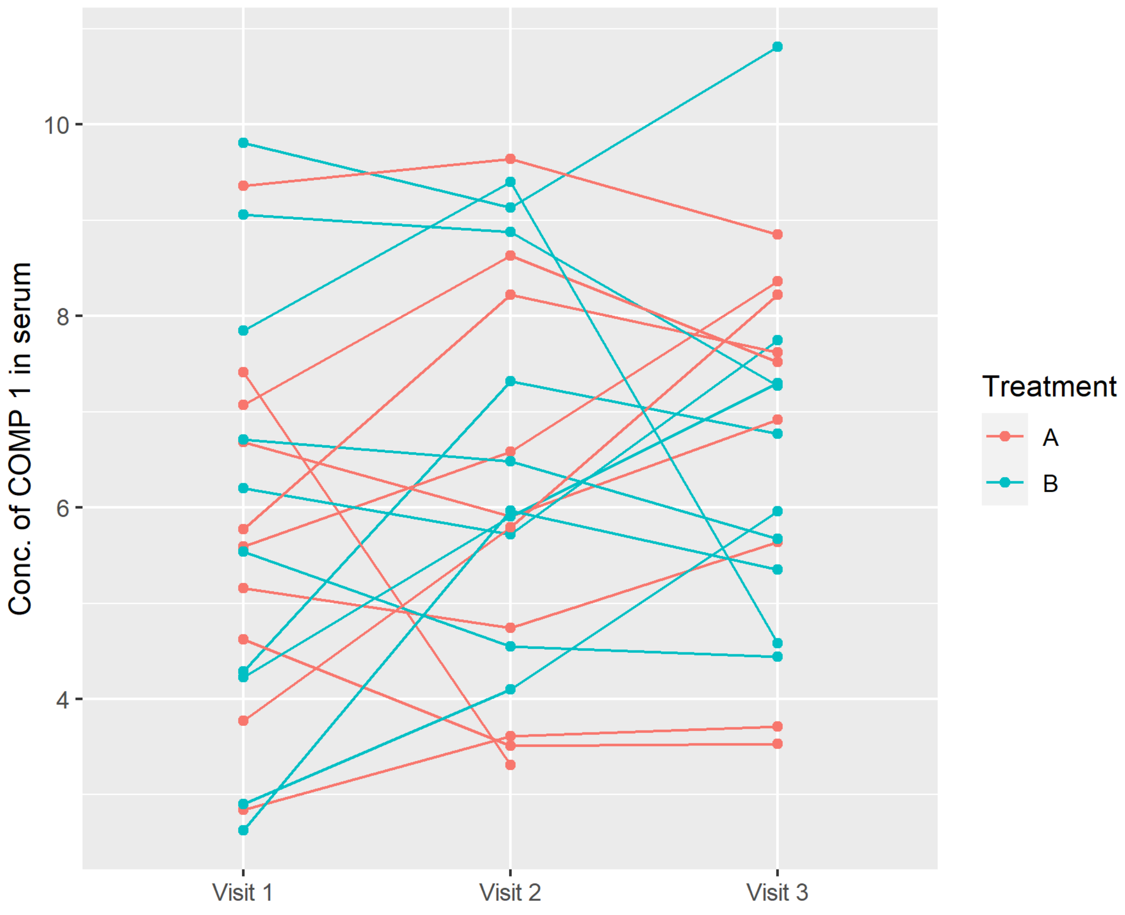

Figure 7. Line chart illustrating individual trajectories for concentration of COMP 1 in serum at visit 1, 2 and 3

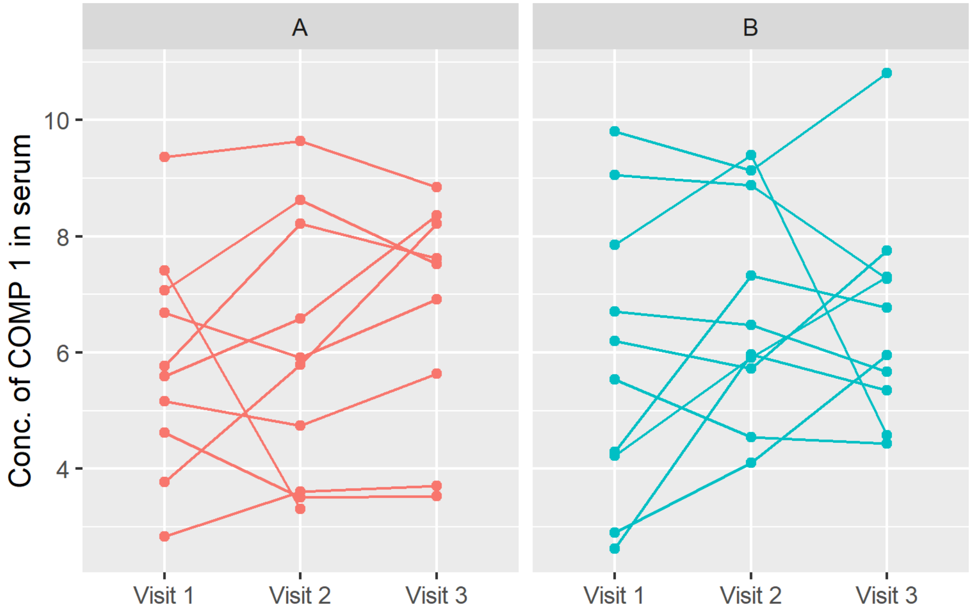

Figure 8. Line chart illustrating individual trajectories for concentration of COMP 1 in serum for each treatment group separately

DS
DS

DocuSign Envelope ID: 06A04576-8AB2-4997-AFCE-75ABF2223EF0

APPENDIX A. TESTS OF NORMALITY ASSUMPTIONS

Table A 1. Tests of normality for Q-score and lameness after flexion test for visit 1, 2 and 3

test	statistic	p.value	method
Q-score v1	0,908	0,058	Shapiro-Wilk normality test
Q-score v2	0,858	0,007	Shapiro-Wilk normality test
Q-score v2	0,872	0,013	Shapiro-Wilk normality test
lameness v1	0,809	0,001	Shapiro-Wilk normality test
lameness v2	0,798	0,001	Shapiro-Wilk normality test
lameness v3	0,522	0,000	Shapiro-Wilk normality test

Table A 2. Test of normality for difference in concentrations between visit 1 and 2 for biomarkers in synovial fluid

test	statistic	p.value	method
COMP1 low log	0,845	0,004	Shapiro-Wilk normality test
BGN 262 low log	0,921	0,103	Shapiro-Wilk normality test

Table A 3. Test of normality for residuals of fitted linear mixed models for the logarithmic values of the concentration of the biomarkers in serum

test	statistic	p.value	method
residuals marginal BGN serum log	0,981	0,480	Shapiro-Wilk normality test
residuals ss BGN serum log	0,966	0,103	Shapiro-Wilk normality test
residuals marginal COMP1 serum log	0,966	0,097	Shapiro-Wilk normality test
residuals ss COMP1 serum log	0,975	0,258	Shapiro-Wilk normality test

DS FB
DS MP

DocuSign Envelope ID: 06A04576-8AB2-4997-AFCE-75ABF2223EF0

APPENDIX B. BASELINE VALUES AND DEMOGRAPHICS

Table B 1. Demographics by group and on total level

	A (N = 10)	*B (N = 10)*	*df (N = 20)*
**Age**			
**min:**	2	2	2
**mean (sd)**	3.50 (1.84)	4.50 (2.37)	4.00 (2.13)
**median (IQR)**	3.00 (2.25, 3.75)	5.00 (2.00, 6.00)	3.00 (2.00, 6.00)
**max:**	8	8	8
**Sex**			
**Mare, n (%)**	2 (20)	1 (10)	3 (15)
**Stallion, n (%)**	4 (40)	4 (40)	8 (40)
**Gelding, n (%)**	4 (40)	5 (50)	9 (45)

Table B 2. Baseline values for biomarkers in serum and synovial fluid by group and on total level

	A (N = 10)	*B (N = 10)*	*Total (N = 20)*
**BGN 262 (low) visit 1**			
**min:**	96.66	55.83	55.83
**mean (sd)**	383.01 (194.62)	238.75 (119.34)	310.88 (173.68)
**median (IQR)**	344.93 (285.42, 527.13)	258.23 (139.63, 312.50)	298.33 (190.81, 385.14)
**max:**	665.66	428.99	665.66
**COMP 1 (low) visit 1**			
**min:**	12.54	2.39	2.39
**mean (sd)**	23.90 (9.95)	20.61 (13.31)	22.25 (11.56)
**median (IQR)**	21.04 (18.36, 29.33)	17.17 (14.93, 24.23)	20.19 (15.47, 25.53)
**max:**	41.75	52.95	52.95
**BGN 262 serum visit 1**			
**min:**	1219.74	1017.24	1017.24
**mean (sd)**	1,526.41 (188.46)	1,477.05 (260.62)	1,501.73 (222.80)
**median (IQR)**	1,527.32 (1,466.58, 1,619.36)	1,523.30 (1,337.56, 1,665.00)	1,523.30 (1,352.12, 1,664.47)
**max:**	1839.04	1856.81	1856.81
**COMP 1 serum visit 1**			
**min:**	2.84	2.63	2.63
**mean (sd)**	5.83 (1.90)	5.92 (2.47)	5.87 (2.15)
**median (IQR)**	5.68 (4.75, 6.97)	5.87 (4.25, 7.56)	5.68 (4.28, 7.16)
**max:**	9.36	9.81	9.81

DocuSign Envelope ID: 06A04576-8AB2-4997-AFCE-75ABF2223EF0

APPENDIX C. FIGURES FOR CONCENTRATION OF BIOMARKERS

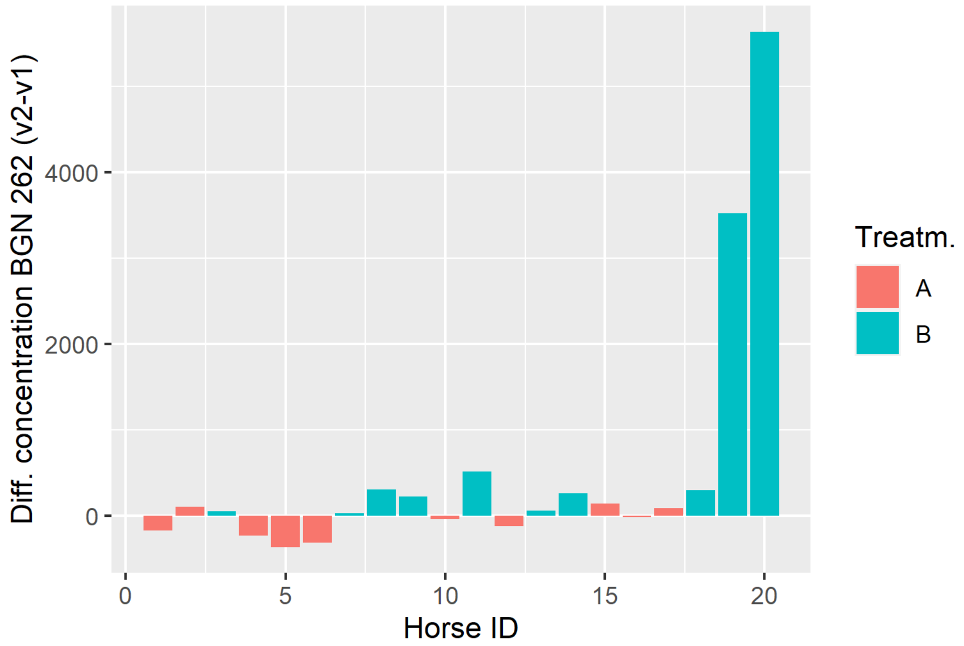

Figure C 1. Differences in concentration of BGN 262 in synovial fluid in the carpal joint (upper or middle joint compartment) that showed the lowest concentration at visit 1

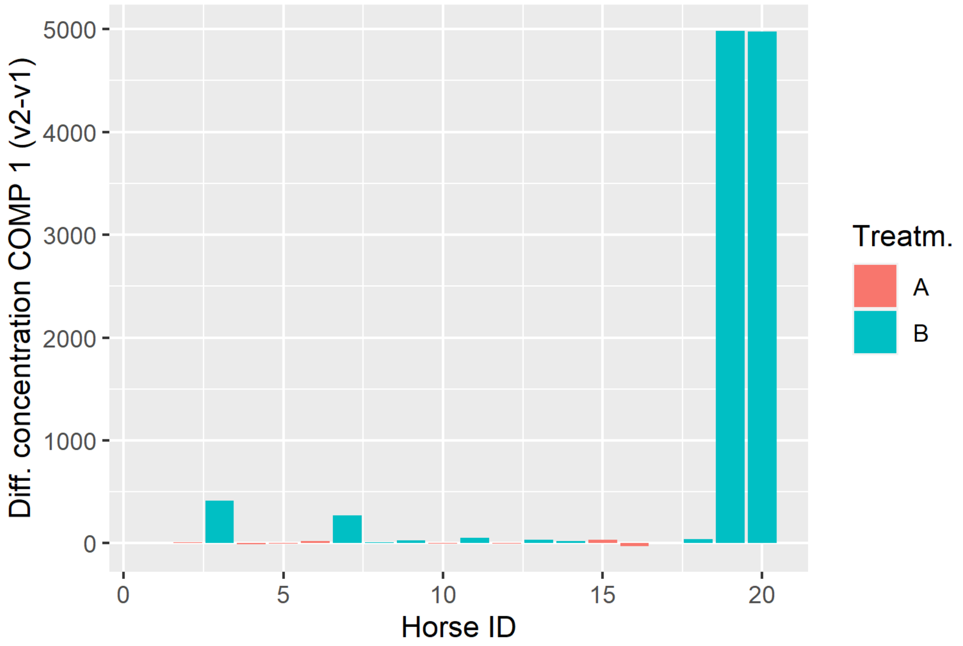

Figure C 2. Differences in concentration of COMP 1 in synovial fluid in the carpal joint (upper or middle joint compartment) that showed the lowest concentration at visit 1

DocuSign Envelope ID: DDF688A5-962F-4AD2-9A99-DBE143A83945

Statistikkonsulterna
JOSTAT & MR SAMPLE AB

Analysis of interview question- how is the horse trotting?

Study title: Intra articular treatment with a new drug combination in comparison with Celeston Bifas in horses with lameness associated with osteoarthritis (OA-252)

**Date:**	**Authors:**
2022-07-15	Magnus Pettersson
	DocuSigned by: Magnus Pettersson 2B47B83A416F4A2...
**Status**:	
Final	

Summary

This report contains results for the subjective assessment by interviewing the trainer, conducted at visits 1 and 4. Data consist of interviews made by veterinarian of the trainer.

In this report only the assessment of trotting is included. The other parameters as mood, appetite and fur quality did not show any differences between visit 1 and 4 and are not included in the report.

These analyses are not described in SAP for the study "*Intra-articular treatment with a new drug combination in comparison with Celeston bifas in horses with lameness associated with Osteoarthritis*" and is an addendum to the analyses presented in report 1 and 2.

DocuSign Envelope ID: DDF688A5-962F-4AD2-9A99-DBE143A83945

TABLE OF CONTENTS

Summary 1

1. Data description and statistical methods 3

1.1 Data description 3

1.2 Missing data 3

1.3 Statistical analysis 4

1.4 Analysis software 4

2. Results 4

2.1 Comparison between treatments at visit 1 4

2.2 Comparison between treatments at visit 4 5

2.3 Comparison between visits 1 and 4 for treatment A 6

2.4 Comparison between visits 1 and 4 for treatment B 6

2.5 Summary of statistical tests 7

DS

DocuSign Envelope ID: DDF688A5-962F-4AD2-9A99-DBE143A83945

1. DATA DESCRIPTION AND STATISTICAL METHODS

1.1 Data description

Data consists of an assessment at visits 1 and 4, respectively, where the trainer has been interviewed regarding the following questions:

The trainer were not given information regarding the selected treatment for the horse.

At visit 1 the trainer was interviewed by the veterinarian (Kristin Abrahamsson-Aurell (KAA)).

The following questions were asked by KAA to the trainer;

1. How is the horse trotting?
2. How is the horse appetite?
3. How is the horse mood?
4. How is the quality of the horse's fur?

The answers were recorded in the journal.

The answers were graded on 1-4 scale according to the following:

1 = bad, 2 = normal, 3 = good, 4 = fantastic.

Interview follow-up after 60 days

Follow-up information was collected two months post-treatment by the veterinarian KAA interviewing the trainers by telephone.

The same questions as at visit 1 was asked:

1. How is the horse trotting?
2. How is the horse appetite?
3. How is the horse mood?
4. How is the quality of the horse's fur?

The answers were recorded in the journal.

The answers were graded on 1-4 scale according to the following:

1 = bad, 2 = normal, 3 = better, 4 = much better.

1.2 Missing data

Two horses in group A were not assessed at visit 4 regarding how the horse was trotting. The trainer couldn't start to train the horse at the time for the interview due to bad winter weather. The horse was therefore given a prolonged rest. The data is treated as missing.

DS
Mp

DocuSign Envelope ID: DDF688A5-962F-4AD2-9A99-DBE143A83945

1.3 Statistical analysis

Data is presented as cross tables for:

1) Comparison between treatments at visit 1
2) Comparison between treatments at visit 4
3) Comparison between visit 1 and 4 for treatment A
4) Comparison between visit 1 and 4 for treatment B

P-values for Fisher's exact tests are presented, where the null hypothesis is that there are no treatment effect at visit 1 and 4, respectively; and that there are no differences between the answers at visit 1 and 4 for each treatment group.

A significance level of 5% is used.

1.4 Analysis software

R (version 4.0.0) has been used for all analyzes.

2. RESULTS

2.1 Comparison between treatments at visit 1

Table 1. Comparison on treatment effect for assessment of trotting at visit 1 according to interview

	Treatment vs Trotting, visit 1				
	Trotting visit 1				
**Treatment**	**1**	**2**	**3**	**4**	**Total**
**A – Drug combination**	5	4	1	0	**10**
**B – Celeston bifas**	7	3	0	0	**10**
**Total**	**12**	**7**	**1**	**0**	**20**

There is no significant difference between the treatment groups, p = 0.650.

DS
Mp

DocuSign Envelope ID: DDF688A5-962F-4AD2-9A99-DBE143A83945

2.2 Comparison between treatments at visit 4

Table 2. Comparison on treatment effect for assessment of trotting at visit 4 according to interview

Treatment vs Trotting, visit 4					
	Trotting visit 4				
**Treatment**	**1**	**2**	**3**	**4**	**Total**
**A – Drug combination**	0	0	4	4	**8**
**B – Celeston bifas**	3	3	3	1	**10**
**Total**	**3**	**3**	**7**	**5**	**18**

There is a significant difference between the treatment groups for the change between visit 1 and 4, p = 0.044 (*).

DS
MP

DocuSign Envelope ID: DDF688A5-962F-4AD2-9A99-DBE143A83945

2.3 Comparison between visits 1 and 4 for treatment A

Table 3. Comparison of assessment at visits 1 and 4 for treatment A (Drug combination) according to interview

	Visit 1 vs Visit 4, Treatment A				
	Trotting visit 4				
**Trotting visit 1**	**1**	**2**	**3**	**4**	**Total**
**1**	0	0	1	3	**4**
**2**	0	0	2	1	**3**
**3**	0	0	1	0	**1**
**4**	0	0	0	0	**0**
**Total**	**0**	**0**	**4**	**4**	**8**

There is no significant difference between the treatment groups, p = 0.486.

2.4 Comparison between visits 1 and 4 for treatment B

Table 4. Comparison of assessment at visits 1 and 4 for treatment B (Celeston bifas) according to interview

	Visit 1 vs Visit 4, Treatment B				
	Trotting visit 4				
**Trotting visit 1**	**1**	**2**	**3**	**4**	**Total**
**1**	2	2	2	1	**7**
**2**	1	1	1	0	**3**
**3**	0	0	0	0	**0**
**4**	0	0	0	0	**0**
**Total**	**3**	**3**	**3**	**1**	**10**

DS
MP

DocuSign Envelope ID: DDF688A5-962F-4AD2-9A99-DBE143A83945

There is no significant difference between the treatment groups, p = 1.

2.5 Summary of statistical tests

The tests conducted in this report is summarised in Table 5.

Table 5. Summary of comparisons for assessment of trotting

Test	p-value
Treatment A vs B, visit 1	0.65
Treatment A vs B, visit 4	0.044
Visit 1 vs 4, treatment A	0.486
Visit 1 vs 4, treatment B	1

DS
MP
